# Comparing Brain and Blood Lipidome Changes following Single and Repetitive Mild Traumatic Brain Injury in Rats

**DOI:** 10.1021/acschemneuro.3c00603

**Published:** 2024-01-05

**Authors:** Alexis N. Pulliam, Eric C. Gier, David A. Gaul, Samuel G. Moore, Facundo M. Fernández, Michelle C. LaPlaca

**Affiliations:** † Coulter Department of Biomedical Engineering, Georgia Institute of Technology/Emory University, Atlanta, GA 30332 USA; ‡ School of Chemistry and Biochemistry, Georgia Institute of Technology, Atlanta, GA 30332 USA; § Petit Institute for Bioengineering and Bioscience, Georgia Institute of Technology, Atlanta, GA 30332, USA

**Keywords:** Traumatic brain injury, lipidomics, blood biomarkers, brain biomarkers, ultra-high performance mass spectrometry

## Abstract

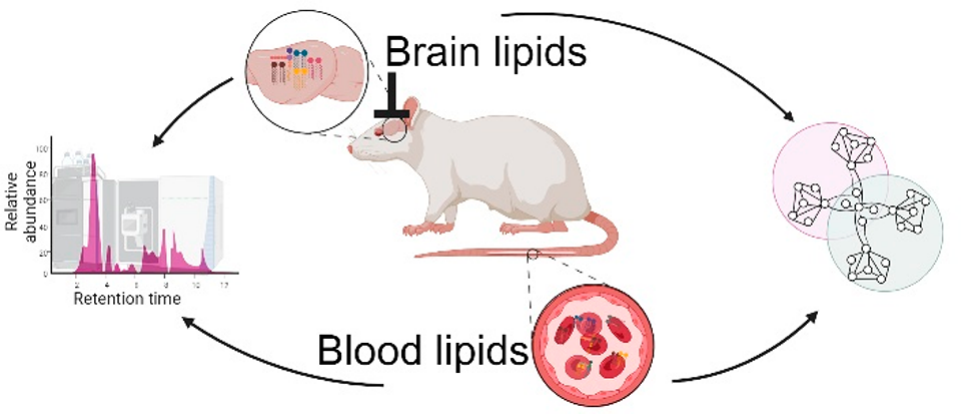

Traumatic brain injury (TBI) is a major health concern in the United States and globally, contributing to disability and long-term neurological problems. Lipid dysregulation after TBI is underexplored, and a better understanding of lipid turnover and degradation could point to novel biomarker candidates and therapeutic targets. Here, we investigated overlapping lipidome changes in the brain and blood using a data-driven discovery approach to understand lipid alterations in the brain and serum compartments acutely following mild TBI (mTBI) and the potential efflux of brain lipids to peripheral blood. The cortices and sera from male and female Sprague–Dawley rats were analyzed via ultra-high performance liquid chromatography–mass spectrometry (UHPLC-MS) in both positive and negative ion modes following single and repetitive closed head impacts. The overlapping lipids in the data sets were identified with an in-house data dictionary for investigating lipid class changes. MS-based lipid profiling revealed overall increased changes in the serum compartment, while the brain lipids primarily showed decreased changes. Interestingly, there were prominent alterations in the sphingolipid class in the brain and blood compartments after single and repetitive injury, which may suggest efflux of brain sphingolipids into the blood after TBI. Genetic algorithms were used for predictive panel selection to classify injured and control samples with high sensitivity and specificity. These overlapping lipid panels primarily mapped to the glycerophospholipid metabolism pathway with Benjamini–Hochberg adjusted *q*-values less than 0.05. Collectively, these results detail overlapping lipidome changes following mTBI in the brain and blood compartments, increasing our understanding of TBI-related lipid dysregulation while identifying novel biomarker candidates.

## 1 Introduction

Traumatic brain injury (TBI) is a neurological disorder resulting from direct or indirect physical loading of the head that translates to the brain, causing transient or persistent cognitive and/or motor impairments.^1,2^ TBI is a leading cause of disabilities in the United States^3^ and a potential risk factor in developing neurodegenerative disorders such as Alzheimer’s disease,^4−6^ Parkinson’s disease,^7,8^ and chronic traumatic encephalopathy (CTE).^6,9^ Primary injury from TBI results from the direct mechanical damage at initial impact, leading to axonal shearing,^10^ and tissue and cellular membrane damage.^6,11^ Secondary injury cascades such as excitotoxicity,^12^ mitochondrial dysfunction,^13^ and lipid peroxidation^14,15^ follow in the acute and chronic phases after injury and contribute to the complexity and heterogeneity of injury manifestation across injury severities.

In the United States, there are approximately 2.5 million TBIs per year with 75–80% considered mild.^1,16^ It is estimated the annual number of mild TBI (mTBI) is much higher due to underreporting.^17,18^ Additionally, many mTBI remain misdiagnosed in the clinical setting due to the reliance on subjective self-reported symptoms.^19^ Therefore, there is a need for quantitative diagnostic aids that are both specific and sensitive to mTBI and can provide objective measures that can be used in conjunction with other clinical tools such as cognitive testing and symptom reporting. Fluid biomarkers for TBI assessment hold promise to translate clinically due to the relative ease of accessibility to biofluids such as blood, saliva, and urine. Additionally, fluid biomarkers may have the ability to track disease progression, which can aid in clinical management.

The FDA has cleared glial fibrillary acidic protein (GFAP) and ubiquitin C-terminal hydrolase L1 (UCHL-1) as the first clinical blood test for TBI to assess the need for CT imaging after brain injury; yet, there remains no blood marker to assess TBI severity. Protein biomarkers such as GFAP,^20,21^ UCHL-1,^21,22^ S100B,^23,24^ tau,^25,26^ phospho-tau,^25^ and NF-L^27^ have been widely investigated in preclinical and clinical TBI studies. These protein biomarkers are large intracellular molecules and can identify severe TBI in both preclinical and clinical studies, but the usefulness has been inconsistent for milder injuries.^28^ Furthermore, large molecules may be diffusion-limited in the brain parenchyma and do not readily cross into the peripheral blood due to minimal damage of the blood brain barrier (BBB) after mTBI.^29^ However, these markers may exit to the peripheral blood through the glymphatic system via bulk flow, which in combination with variable BBB opening may explain inconsistencies in TBI studies.^30,31^ Consequently, lipids are attractive molecules to investigate as a diagnostic aid for mTBI due to their relatively small size and relative ease of diffusion across an intact BBB.

Lipids as biomarkers have been underexplored in the field of TBI research. Lipids are small biomolecules that make up approximately 50% of the brain’s dry weight,^32^ 70–85% of the total composition of the myelin sheath,^33^ and 50–60% of the total mass of cellular membrane.^34^ Because of the high abundance of lipids in the brain and the susceptibility for oxidative damage, investigating lipids in preclinical models may both help us to understand TBI pathophysiology and identify novel translatable biomarker candidates. The field of lipidomics has evolved to include high-resolution analytical chemistry techniques and bioinformatics tools, allowing detailed investigation of lipid changes in neurological diseases.^35^ Studies using mass spectrometry have shown an increase in pro-inflammatory lipids such as bioactive lipids and lysophospholipids, which is consistent with post-TBI neuroinflammation.^36−38^ Phospholipids are major constituents of the plasma membrane, and because TBI has been shown to cause membrane disruption,^11^ it follows that phospholipid dysregulation may be part of the injury process. Previous groups have reported significant changes of phospholipid species in single and repetitive TBI models.^39−41^ Specifically, an increase in total phospholipids in the cortex hippocampus was demonstrated in the chronic phase post-TBI.^41^ Previous studies from our group have identified a 26-serum lipid panel that differentiates moderate TBI and sham control 3- and 7-days post injury.^36^ We saw a significant decrease in some phospholipid species from the candidate biomarker panel. Additionally, we have shown robust serum panels that differentiate mTBI and sham rats in both sexes.^42^ These lipids were mapped onto various pathways such as sphingolipid signaling and necroptosis. There have been multiple studies that seek to understand lipid changes and investigate lipids as biomarkers for TBI. However, no one to our knowledge has investigated global lipidome changes in both the serum and brain after single and repetitive mTBI. The objectives of this study were to identify lipids observed in both the brain and serum, investigate abundances of these lipids in the brain and serum compartments, and map pathways of these lipids that distinguish between TBI and control to begin to understand the potential lipid efflux from the brain to the peripheral blood.

## 2 Results

### 2.1 Descriptive Lipidome Changes in Detected Lipids in the Brain and Blood Compartments after mTBI

**Figure 1:**
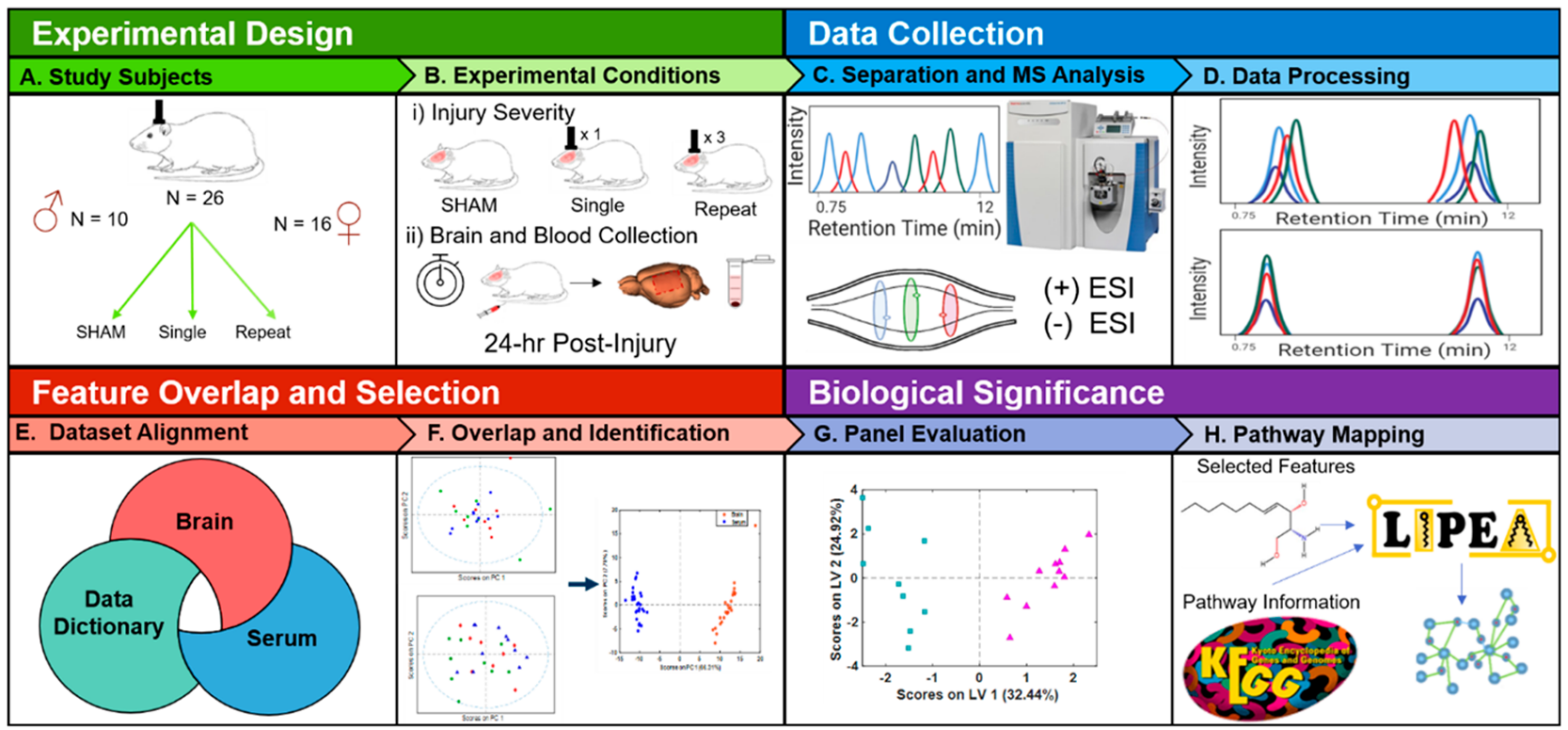
Overview of the workflow from study design to interpretation. (A) Experimental groups included males (*n* = 14) and females (*n* = 18). Rats were assigned to a sham control group that received no injuries (*n* = 11), a single impact group that received one impact (*n* = 10), or a repeat impact group that received three separate impacts (*n* = 11). (B) Injury groups received closed head impacts, and the blood and brains were collected at 24 h postinjury. Lipids were extracted with homogenization in isopropanol. (C) Samples were analyzed in random order with high-resolution LC-MS. (D) Spectral alignment, peak detection, isotope and adduct grouping, gap filling, and drift correction were accomplished using Compound Discoverer v.3.0. (E) Lipids detected in the brain and serum data sets were selected and annotated by spectra matching to in-house databases. (F) PCA score plot of brain and serum data sets. (G) Features selected by machine learning algorithms were combined to create an oPLS-DA model. (H) Annotated compounds selected in the models were imported into LIPEA to explore biological pathways altered following TBI.

To compare lipidome changes detected by UHPLC-MS in the cortex and serum after mTBI, we reduced features based on the overlap between the compartments (Figure S1). There were 14,909 features detected in the cortex data set and 14,119 features in the serum data set. The results indicated prominent separation along PC1 between male and female serum samples; however, there was more overlapping of sexes in the cortex than in the serum (Figure S1A and B). Additionally, there was more clustering among injury groups along the diagonal of PC1 and PC2 of the serum that was not seen in the brain data sets (Figure S1C and D). These results suggest greater changes in the serum due to injury at 24 h. To further reduce the features, the cortex and serum data sets were then concatenated with an in-house data dictionary to identify lipid species that overlap in each compartment based on exact mass, chemical formula, and retention time. There were 318 features in the cortex and, using parallel identification, 349 in the serum data sets with tentative annotations (Figures S2). Isotopic features remained in the initial analysis and were later processed to compare class changes, identify candidate lipid biomarkers, and pathway analysis of lipid panels (Figure 1).

**Figure 2:**
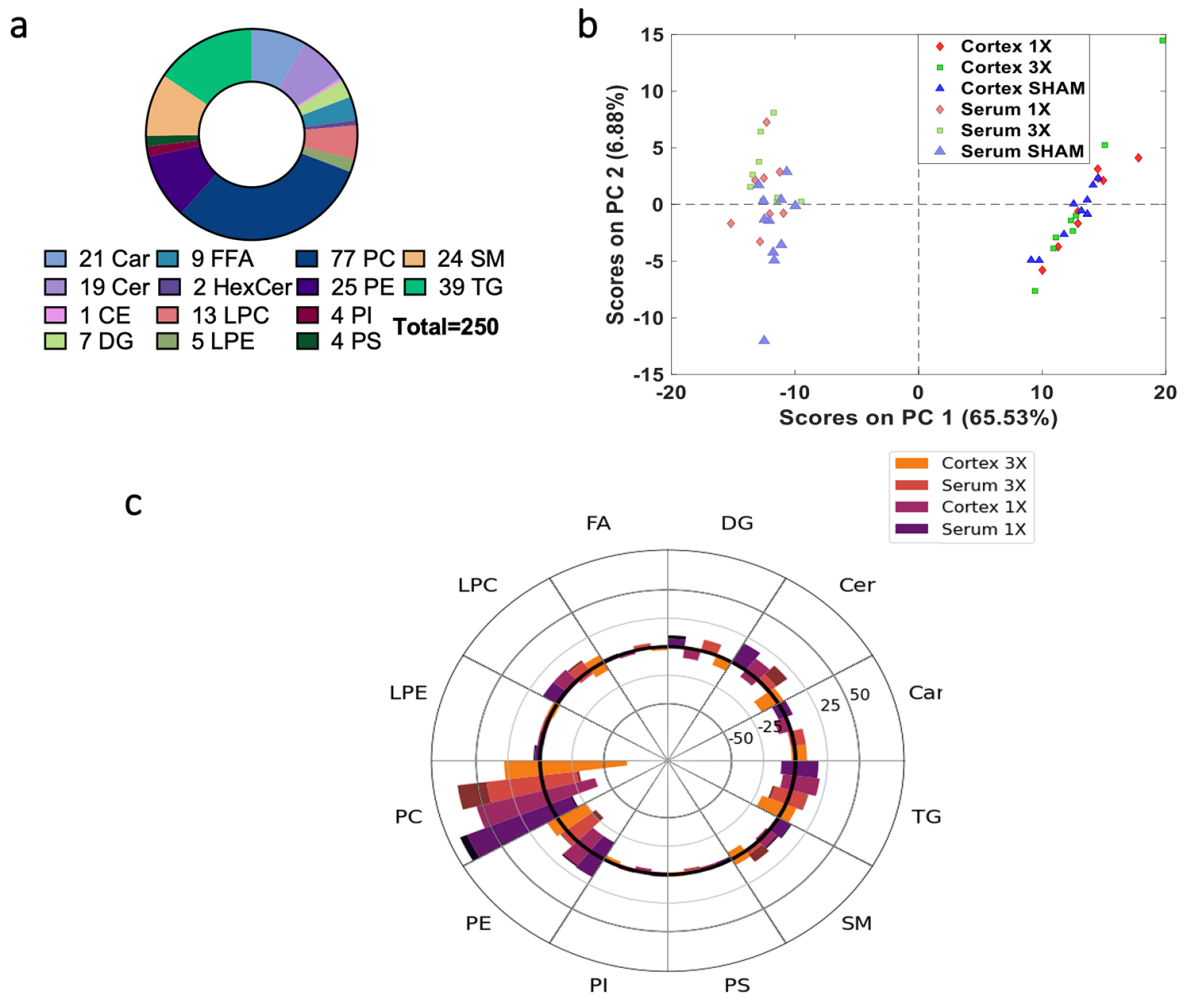
Annotated features observed in the brain and serum compartments. (A) Pie chart of number of annotated lipids that overlapped in the cortex and serum data sets. (B) PCA score plot of 250 annotated lipids that overlap in the brain and serum compartments. Data show clear separation along PC1 of the brain and serum metabolites. (C) Spider plot of annotated features in both brain and serum data sets based on lipid subclasses depicts the number of lipids that either increased or decreased due to injury in each compartment. Dark colored bars signify number of significant features between injury severity and sham. Welch’s *t* test *p* < 0.05. Abbreviations: Car – acyl carnitines, CE – cholesteryl esters, Cer – ceramides, DG – diacylglycerols, FFA – free fatty acids, LPC – lysophosphatidylcholine, LPE – lysophosphatidylethanolamine, PC – phosphatidylcholine, PE – phosphatidylethanolamine, PI – phosphatidylinositol, PS – phosphatidylserine, SM – sphingomyelin, TG – triacylglycerols.

The overlapping data sets were further aligned by the summation of isomers, which left 250 tentatively annotated features in both data sets that show separation of sex along PC2 for the serum and slight separation of injury severity along the diagonal of PC2 (Figure S3). These results above indicate a prominent separation between sexes; however, male and female samples were grouped for analysis to understand overall lipidome changes in overlapping lipids detected between the brain and serum compartments due to TBI. Three metabolites belonging to the cholesterol ester (CE) and hexosylceramide (HexCer) lipids were removed for lipid subclass analysis due to limited identification. The 247 overlapping, tentatively identified metabolites in the cortex and serum compartments were members of the acyl carnitine (Car), ceramide (Cer), diacylglyceride (DG), free fatty acid (FFA), lysophosphatidylcholine (LPC), lysophosphatidylethanolamine (LPE), phosphatidylcholine (PC), phosphatidylethanolamine (PE), phosphatidylinositol (PI), phosphatidylserine (PS), triacylglyceride (TG), and sphingomyelin (SM) subclasses (Figure 2A). Most of the overlapping lipids were PCs, a prominent constituent of the phospholipid cellular membrane. Expectedly, the PCA score plot of the 247 overlapping, annotated lipids showed prominent separation among the tissue and fluid compartments along PC1 (Figure 2B). Qualitatively, the serum groups separated more than the cortex groups, which suggests more variability in the serum. This result was illustrated in the broader spread of the serum sham control cluster compared to the cortex sham control cluster. Most of the 3X serum samples clustered in the upper left quadrant, and the 3X cortex samples clustered in the lower right quadrant; the 1X and serum samples clustered near the midline of the respective quadrants (Figure 2B).

#### 2.1.1 Comparison between Injury Severity in the Brain

To compare changes due to injury in the cortex and serum compartments, we evaluated the lipid subclasses of injury groups relative to the sham control for both single (1X) and repetitive (3X) TBI control. The spider plot illustrates that 93 lipids increased (greater than 1) or 154 lipids decreased (less than 1) after mTBI relative to sham control (Figure 2A). When injury severity was compared among the cortex 1X and 3X groups, the SM and Cer groups had inverse relationships between the cortex 1X and 3X groups. There were 19 Cer and 24 SM detected in the data sets. There were 13 ceramides that decreased and 6 that increased due to injury in the cortex 3X group. However, only 2 decreased and 17 increased in the cortex 1X group. We observed a similar trend in the SM subclass where more lipids decreased due to injury in the cortex 3X groups compared with the cortex 1X group.

DG, PC, PE, and TG had similar trends; for example, DG decreased in both groups. Interestingly, the cortex 3X group only had 1 statistically significant lipid, PS(36:1) *p* < 0.05 (Figure 2C and Table 1), while the cortex 1X group had 6 lipids with a *p*-value < 0.05: Cer(d18:1/16:0), PS(36:1), SM(d36:0), SM(d36:1), SM(d38:2), and SM(d39:1) (Figure 2C and Table 1). A summary heatmap of *p*-values is shown in the Supporting Information (Figure S4). Also, the lipids decreased in the cortex 3X group relative to the sham control in the FFA, LPC, PC, and TG subclasses (Figures 2C).

**Table 1:** List of Statistically Significant *p*-Values and Respective Fold Change Values Relative to the Sham Control^a^

			Injury Severity							
			Cortex 1X vs sham		Cortex 3X vs sham		Serum 1X vs sham		Serum 3X vs sham	
Class	Subclass	Name	*p*-values	Log2(FC)	*p*-values	Log2(FC)	*p*-values	Log2(FC)	*p*-values	Log2(FC)
Fatty Acid	Carnitine	Car(24:0)	0.635	–0.794	0.510	–0.590	0.696	–0.303	0.003**	0.022
		Car(22:0)	0.696	0.026	0.365	–0.225	0.587	0.059	0.013*	0.419
		Car(19:1)	0.172	–0.216	0.463	0.092	0.114	0.766	0.023*	0.879
	Free Fatty Acid	FA(24:1)	0.976	0.114	0.337	0.306	0.121	1.224	0.021*	2.436
Glycerolipid	Diacylglyceride	DG(40:7)	0.756	–0.192	0.870	–0.088	0.028*	0.595	0.005**	0.684
		DG(38:5)	0.575	–0.111	0.827	–0.052	0.0002***	0.317	0.007**	0.747
		DG(36:4)	0.784	–0.223	0.716	–0.020	0.163	0.291	0.014*	1.181
		DG(38:4)	0.923	–0.058	0.659	–0.138	0.429	0.045	0.015*	0.331
		DG(40:6)	0.104	–0.362	0.380	0.079	0.027*	0.722	0.126	0.657
	Triacylglyceride	TG(46:0)	0.121	0.259	0.282	0.139	0.507	–0.284	0.011*	–0.901
		TG(48:1)	0.302	0.145	0.370	0.051	0.850	–0.302	0.025*	–0.783
		TG(46:1)	0.333	–0.049	0.142	0.913	0.432	–0.508	0.027*	–1.097
Phospholipid	Lysophosphatidylcholine	LPC(20:4)	0.554	0.013	0.616	0.703	0.088	0.237	0.033*	0.208
		LPC(18:0)	0.233	0.106	0.699	–0.070	0.243	0.117	0.044*	0.226
		LPC(14:0)	0.881	–0.160	0.362	–0.201	0.349	0.269	0.044*	–0.382
	Phosphatidylcholine	PC(42:4)	0.230	0.217	0.352	–0.275	0.040*	0.289	0.003**	0.375
		PC(31:1)	0.844	–0.159	0.684	–0.130	0.924	–0.020	0.006**	–0.853
		PC(37:4)	0.663	–0.020	0.652	–0.031	0.070	0.286	0.008**	0.407
		PC(42:9)	0.392	0.447	0.425	0.062	0.094	0.389	0.009**	0.665
		PC(42:5)	0.226	0.301	0.430	–0.256	0.078	0.240	0.010*	0.342
		PC(38:1)	0.177	0.138	0.943	–0.018	0.078	0.429	0.022*	0.517
		PC(40:4)	0.303	–0.012	0.202	–0.212	0.224	0.567	0.023*	0.715
		PC(38:6)	0.491	–0.105	0.368	0.041	0.049*	0.328	0.023*	0.218
		PC(37:2)	0.489	0.102	0.964	–0.304	0.649	–0.366	0.028*	–1.039
		PC(37:1)	0.577	0.029	0.503	0.062	0.014*	0.387	0.029*	0.452
		PC(38:4)	0.355	0.028	0.216	–0.131	0.190	0.270	0.032*	0.387
		PC(44:4)	0.466	–0.108	0.962	–0.023	0.208	0.243	0.032*	0.292
		PC(30:0)	0.110	0.080	0.675	–0.154	0.857	0.162	0.042*	–0.274
		PC(39:4)	0.671	–0.139	0.991	–0.125	0.972	–0.086	0.047*	0.146
		PC(36:1)	0.235	0.091	0.788	0.004	0.096	0.403	0.048*	0.424
		PC(35:0)	0.693	–0.034	0.906	–0.141	0.005*	0.337	0.067	0.176
		PC(41:2)	0.694	0.345	0.577	–0.132	0.029*	0.526	0.693	0.152
		PC(O-36:5)	0.388	0.248	0.390	–0.128	0.315	0.125	0.007**	0.320
		PC(O-40:7)	0.859	–0.038	0.225	0.013	0.182	0.387	0.007**	0.994
		PC(O-38:7)	0.400	0.107	0.864	0.007	0.510	0.207	0.011*	0.648
		PC(O-38:4)	0.355	0.028	0.216	–0.131	0.215	0.191	0.020*	0.227
		PC(O-34:2)	0.654	0.059	0.755	–0.027	0.077	0.230	0.031*	0.225
		PC(O-40:6)	0.849	0.121	0.225	0.159	0.347	0.171	0.032*	0.44
		PC(O-36:4)	0.617	0.143	0.993	–0.065	0.391	0.099	0.037*	0.147
	Phosphatidylethanolamine	PE(36:3)	0.712	–0.012	0.983	–0.117	0.099	–0.132	0.006**	–1.063
		PE(O-42:7)	0.364	–0.282	0.602	–0.223	0.053	0.352	0.012*	0.405
	Phosphatidylinositol	PI(38:4)	0.615	–0.074	0.808	–0.012	0.160	0.166	0.008**	0.494
	Phosphatidylserine	PS(36:2)	0.579	–0.119	0.205	0.118	0.255	–0.308	0.009**	–1.104
		PS(36:1)	0.038*	0.190	0.049*	0.178	0.657	–0.119	0.713	–0.089
Sphingolipid	Ceramide	Cer(d44:1)	0.855	–0.032	0.993	–0.164	0.122	0.401	0.009*	0.869
		Cer(d42:1)	0.408	0.002	0.943	–0.224	0.249	0.197	0.013*	0.416
		Cer(d44:2)	0.895	0.008	0.612	–0.230	0.432	0.278	0.017*	0.569
		Cer(d43:2)	0.532	0.220	0.396	0.124	0.318	0.149	0.019*	0.364
		Cer(d40:1)	0.853	0.011	0.294	–0.152	0.186	0.351	0.020*	0.697
		Cer(d40:1)	0.222	0.134	0.641	–0.126	0.209	0.489	0.020*	0.771
		Cer(d40:2)	0.137	0.181	0.624	0.034	0.350	0.371	0.024*	0.707
		Cer(d41:1)	0.262	0.250	0.513	–0.019	0.088	0.590	0.042*	0.713
		Cer(d41:0-OH)	0.032*	0.241	0.457	–0.058	0.094	0.445	0.153	0.270
	Sphingomyelin	SM(d42:1)	0.432	–0.030	0.607	–0.015	0.179	0.207	0.000*	0.347
		SM(d41:1)	0.140	0.196	0.853	0.051	0.052	0.712	0.001*	0.852
		SM(d41:2)	0.103	0.511	0.450	0.001	0.049*	0.403	0.001*	0.607
		SM(d36:3)	0.377	0.193	0.584	–0.438	0.135	0.473	0.002*	1.080
		SM(d35:0)	0.230	0.191	0.970	–0.141	0.008*	0.646	0.002*	0.978
		SM(d39:1)	0.019*	0.459	0.366	0.166	0.075	0.694	0.003*	0.906
		SM(d44:3)	0.511	0.204	0.474	0.045	0.071	0.576	0.003*	0.796
		SM(d35:1)	0.253	0.024	0.912	–0.072	0.173	0.152	0.003*	0.577
		SM(d40:2)	0.229	0.101	0.733	0.003	0.174	0.530	0.006*	0.974
		SM(d44:1)	0.690	0.036	0.756	–0.347	0.075	0.187	0.007*	0.396
		SM(d36:2)	0.999	0.027	0.612	0.036	0.503	0.117	0.010*	0.786
		SM(d36:1)	0.008*	0.170	0.820	–0.003	0.436	0.547	0.028*	1.325
		SM(d38:2)	0.031*	0.135	0.789	–0.143	0.136	0.524	0.032*	0.736
		SM(d40:0)	0.203	0.383	0.671	–0.096	0.200	0.390	0.032*	0.409
		SM(d40:1)	0.273	0.716	0.850	0.108	0.187	0.548	0.033*	0.410
		SM(d34:2)	0.174	0.182	0.842	–0.383	0.259	0.139	0.033*	0.269
		SM(d36:0)	0.011*	0.418	0.871	–0.116	0.148	0.650	0.065	1.176

a Significance was defined as *p* < 0.05 (*) or *p* < 0.01 (**), *p* < 0.001 (***), or *p* < 0.0001 (****).

#### 2.1.2 Comparison between Injury Severity in the Blood

Comparison between the serum 1X and 3X groups showed similar trends among all lipid subclasses except PE and TGs, where more lipids decreased in the serum 3X groups relative to the sham control compared to the serum 1X group. We observed an increase in Cer and SM lipid subclasses in both serum 1X and 3X groups. There were 9 Cer and 16 SM that significantly increased due to injury in the serum 3X group, and only 2 SM significantly increased in the cortex 1X relative to sham control.

When surveying statistically significant changes due to mTBI relative to sham control, there were 66 lipids (*p*-value < 0.05) in the serum 3X and 18 lipids (*p*-value < 0.05) in the serum 1X (Figure 2C and Table 1). This result further demonstrates a profound injury effect in the serum. All of the lipid subclasses increased due to injury relative to the sham control in the serum, except for the PEs and TGs, where over half of the metabolites decreased due to injury. Expectedly, there were more statistically significant changes in the serum 3X group compared to those in the serum 1X group, which points to a graded injury difference.

#### 2.1.3 Comparison between the Brain and Blood Compartments

The results of our brain and blood analyses show some juxtaposition when comparing compartments. Interestingly, we observed an inverse relationship in the Cer, SM, and DG subclasses, with increases in the serum 3X and decreases in the cortex 3X injury groups relative to the sham control. We observed similar trends in the Car, FFA, PE, and TG subclasses when comparing the cortex and serum compartments (Figure 2C); these metabolites increased in both the serum 3X and cortex 3X groups relative to sham. When comparing the cortex 1X and serum 1X groups, we observed a yin-yang relationship between Car, DG, and FFA subclasses, and similar trends were observed in Cer, LPC, LPE PC, PE, PI, PS, SM, and TG. Some of the lipid subclasses were reflective in the brain and serum compartments. From these results, we aimed to examine quantitative changes due to the injury severity in the brain and serum compartments.

### 2.2 Univariate Analysis of Overlapping Brain and Blood Lipid Classes

#### 2.2.1 Quantitative Changes in the Sphingolipid Class

**Figure 3:**
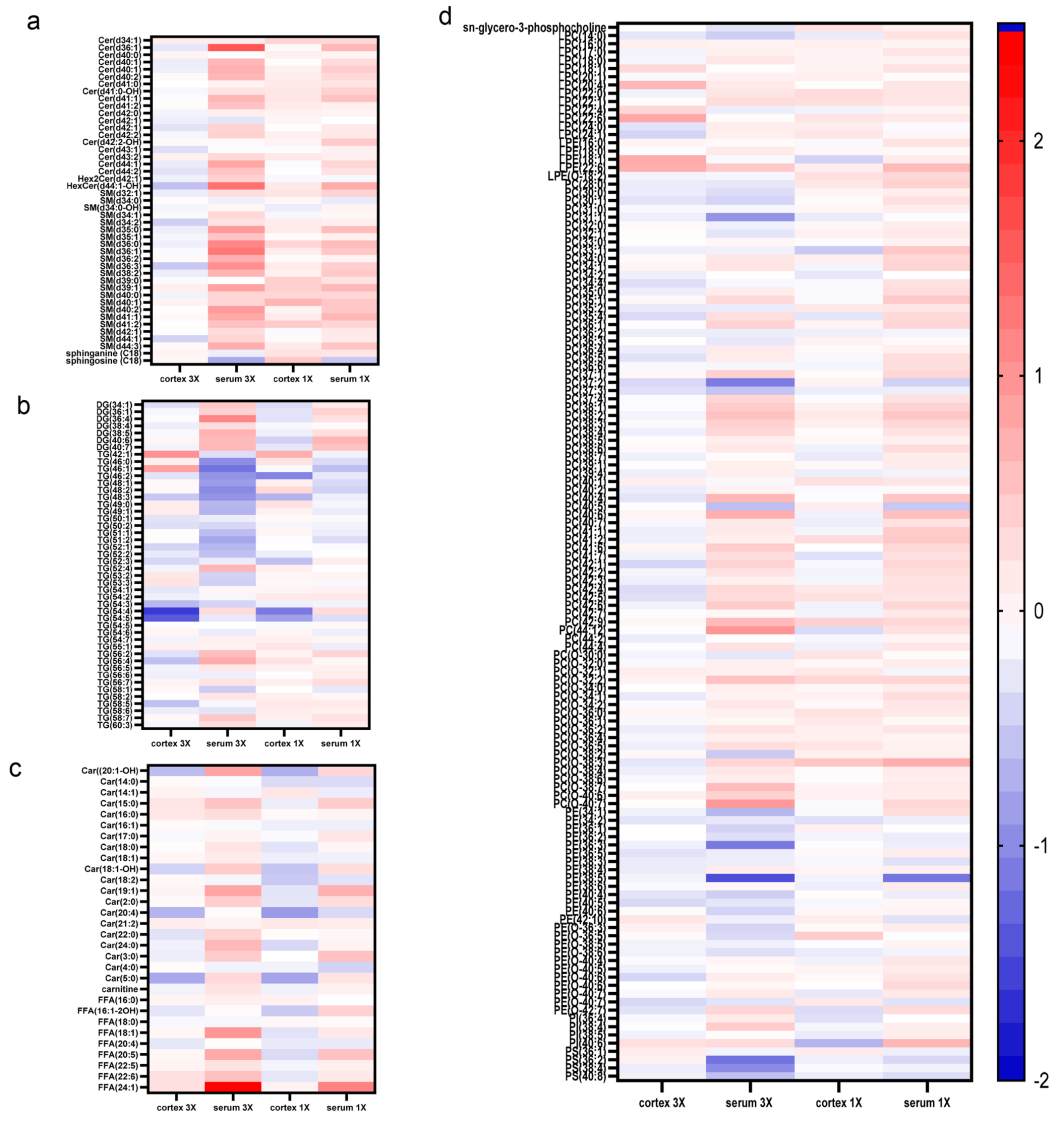
Heatmap of fold change values of the lipid classes. Fold change values of overlapping lipids injury relative to sham control. (A) Sphingolipids. (B) Glycerolipids. (C) Free Fatty Acids. (D) Phospholipids.

The overlapping 249 lipids, omitting the 1 cholesterol ester species and including the 2 hexosylceramides into the analysis of lipid classes, were compared based on compartment and injury severity for a more quantitative examination of the sphingolipid, glycerolipid, fatty acid, and phospholipid classes (Figure 3). The median values of the injured relative to sham control groups were used to compare the lipid subclass and class changes between the brain and serum compartments and for injury severity within the same compartment (Figure 3). The sphingolipid class (Cer and SM) showed interesting results, with most species increased in the injured serum samples relative to the sham control (Figure 3A). There were less pronounced changes in the cortex 1X and 3X groups. However, there were more changes in the brain sphingolipids compared to the other lipid classes. The 3X serum samples had higher fold change values than the serum 1X samples. This result suggests an injury response in this class. HexCer(d18:1_26:0-OH) and HexCer(d20:1_24:0-OH) decreased in the cortex 3X and increased in the serum 3X samples. The SM class decreased in the cortex 3X and increased in the cortex 1X samples. Sphingosine decreased by 151% between the serum 1X and 3X groups. The data illustrate more notable changes in the serum samples compared to the cortex in the acute phase, which may be a result of differential temporal changes or downstream responses in extracranial organ systems (Figure 3A–D).

#### 2.2.2 Quantitative Changes in the Glycerolipid Class

There were notable changes due to mTBI in the glycerolipid subclass (Figure 3B). The heatmap illustrates DGs increase in the serum compartments and either decrease or exhibit negligible changes in the brain when comparing between the compartments (Figure 2C and 3B). When comparing changes due to injury severity of the DG subclass, the data show a graded difference between the 1X and 3X groups (Figure 2C and 3B). For example, DG(16:0_18:1) increased by 95% from cortex 1X to cortex 3X and increased by 244% from serum 1X to 3X. The TG subclass demonstrated a different trend where most of the lipid species decreased or negligibly changed in the cortex and serum due to injury (Figure 3B). However, a few metabolites had different trends due to injury in the cortex and serum compartments. For example, (TG 18:0_18:0_20:4) decreased in the cortex 3X and increased in the serum, while TG(42:1) increased in the cortex and decreased in the serum. Comparing injury severity in the brain, TG(18:1_18:1_18:2) and TG(18:1_18:2_18:2) decreased with high fold change values between cortex 3X and 1X groups by 144% and 173%, respectively (Figure 3B). This result further supports that some metabolites may indicate graded injury changes. Approximately ∼50% of the TG class decreased in the 3X serum with fold change values greater than 1.8; however, there were more negligible fold changes in the serum 1X group compared to serum 3X. Arachidonic acid-containing glycerolipids, DG(16:0_20:4_0:0), DG(18:1_20:4_0:0), and (TG 18:0_18:0_20:4), increased in the serum, but FFA(20:4) had minimal changes (Figure 3A and 3B).

#### 2.2.3 Quantitative Changes in the Fatty Acid Class

There was a general trend in the fatty acid class, where the lipids decreased due to injury in the cortex and increased in the serum (Figure 3C). This was seen for Car(20:1-OH), Car(18:1-OH), Car(22:0), FFA(18:1), and FFA(20:5), although the fold change values were relatively low. These results in fatty acids decreasing in the brain and increasing in the serum suggest different patterns due to injury in the acute phase. When comparing lipid changes based on injury severity in the fatty acid class, there were relatively minimal changes from injury, except for FFA(24:1), which had a high fold change value in the serum 1X and 3X groups. FFA(24:1) increased by 199% between serum 3X and 1X groups relative to sham control and 268% between the cortex 3X and 1X groups (Figure 3A). There was a 710% increase and 874% decrease in Car(22:0) between serum 3X and 1X, and brain 3X and 1X, respectively. FFA(24:1) and Car(22:0) demonstrated a graded change with the injury severity.

#### 2.2.4 Quantitative Changes in the Phospholipid Class

Our work also demonstrates changes in the phospholipid class; there were more extensive alterations due to injury in the serum 3X group than the cortex 1X, cortex 3X, and serum 1X groups relative to sham controls (Figure 3D). When comparing the LPE subclass, there was an increase of LPE(22:6) in the cortex 3X and serum groups with high fold change values greater than 1.5. When injury severity in the brain was compared, LPE(18:1) increased by 233% between the cortex 3X and 1X groups. PCs were the most abundant species detected by LCMS and had the most overlapping metabolites in the cortex and serum compartments (see Figure 2A). PC(33:1) had a fold change value greater than 1.5 in the cortex 1X and serum 1X groups. There was a decrease of 283% PC(37:2) between the serum 1X and 3X samples. In addition, 1/3 of the PCs increased in the serum samples. PEs were the second most annotated lipid (Figure 2A). PE(34:1), PE(36:3), PE(18:1_20:4), PE(18:0_22:4), and PE(40:6) decreased in the cortex and serum samples with high fold change values (Figure 3D). There were minimal changes due to injury in the PI species. PI(18:0_22:6) decreased in the cortex 1X and increased in the serum 1X groups. The PS metabolites had large changes in the serum 3X group and milder changes in the cortex 1X. The cortex had overall increases relative to sham control (see Figure 2B) albeit with small quantitative changes (Figure 3C).

### 2.3 Semiquantification of Abundances in the Brain and Blood Compartments

**Figure 4:**
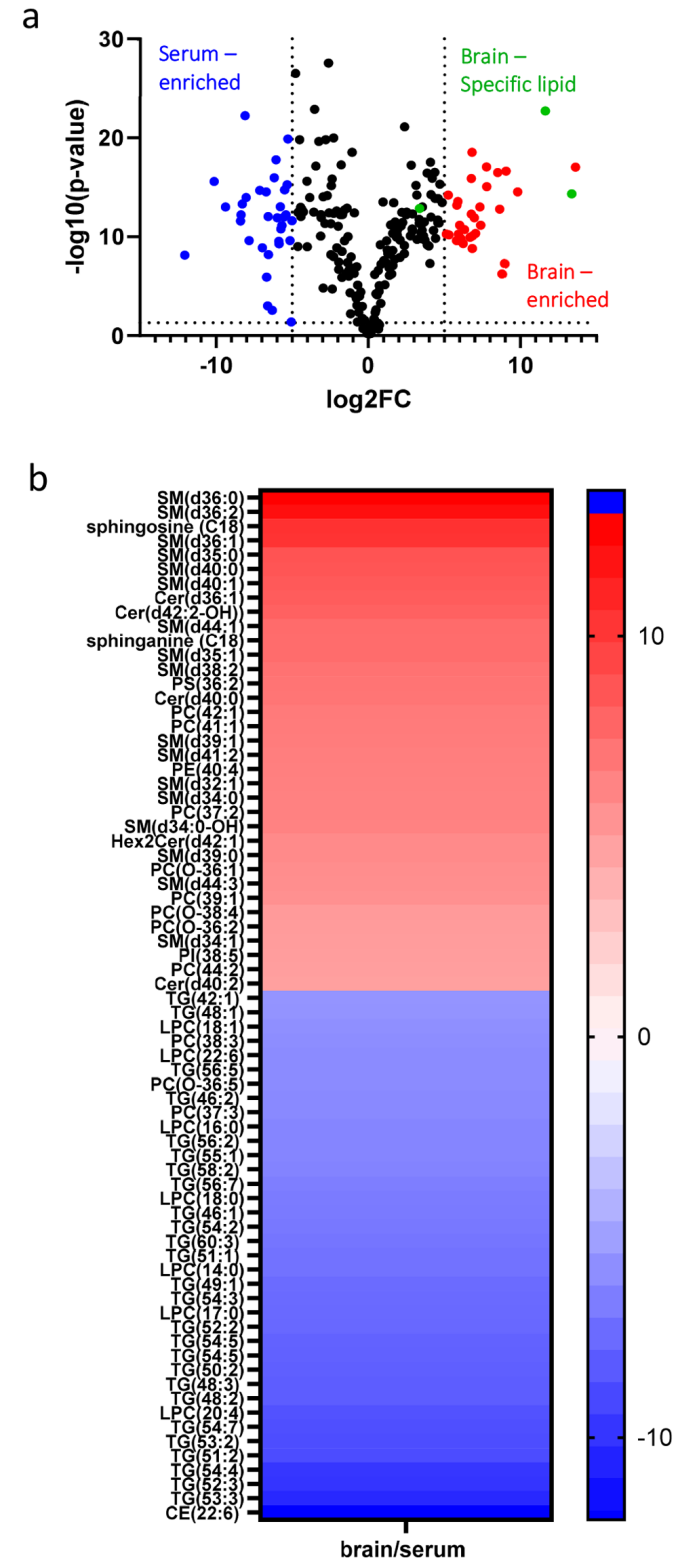
Abundances of overlapping lipids in the cortex and serum. (A) Volcano plot of cortex relative to serum abundances after mTBI. Red dots denote enriched in the brain, blue dots denote enriched in the serum, and green dots denote brain-specific lipids. (B) Heatmap of lipids that are either enriched in the cortex or serum based on log2(FC) of 5 between brain and blood samples. FFA class was removed from analysis due to limitations in the lipid internal standard.

To further explore brain and serum lipidome changes after mTBI, we investigated lipids that were enriched in either the cortex or serum by converting the peak area with standards to obtain the absolute abundance. The LPC standard was used to convert the Car, and PC standard was used to convert Cer due to the chemical properties, and the other standards corresponded to the lipid subclasses. The FFA subclass was removed from analysis because there was no fatty acid internal standard. This approach may lead to understanding of brain lipid efflux to the peripheral blood. Some overlapping metabolites had an abundance over 5-fold in either the brain or serum compartments (Figure 4). The volcano plot illustrates the cortex-to-serum ratio where a positive fold change represents lipids enriched in the brain and a negative fold change represents lipids enriched in the serum (Figure 4A). To further explore brain and blood lipids in our data set, we investigated brain-specific lipid behavior in the brain and the serum. PC(34:0) was previously shown to be a brain-specific lipid;^43^ in our work, there was a 3-fold greater abundance in the brain compared to the serum (Figure 4A) and no statistically significant changes in the serum due to TBI (see Figure S4). However, PC(34:0) did not make the fold change criteria in our data set as a brain-specific lipid, and our results suggest that this lipid may not have been released from the brain to the serum. Twenty-four sphingolipids in our data set are highly enriched in the brain and none in the serum. Specifically, SM(d36:1) and SM(d36:2) were shown to be brain-specific lipids in our data set, which is consistent with the literature that reports a relatively higher abundance of these lipids in the brain and very low abundance in other tissues and plasma.^44^ In our study, we saw these lipids highly enriched in the brain over 10-fold. SM(d36:1) was significantly increased in cortex 1X samples and serum 3X group relative to sham controls (Table 1). SM(d36:2) was significantly increased in the serum 3X relative to sham control. These results suggest that these brain-specific SM species may efflux from the brain to the peripheral blood following TBI.

CE(22:6) was 12-fold greater in the serum than in the brain, which may result from circulating lipoproteins in the bloodstream^45^ (Figure 4B). Some PC and PE species, PC(42:1), PC(41:1), PE(40:4), PC(37:2),PC(O-36:1), PC(39:1), PC(O-38:4), PC(O-362), and PC(44:2), were highly enriched in the brain compared to the serum, which is likely due to the enrichment of phospholipids in the cellular membranes in the neurons and glial cells of the brain (Figure 4B). Although, we assume that the brain-enriched phospholipids in our data set are not necessarily brain-specific lipids due to their presence in other tissues.^43,46,47^ However, LPCs were more abundant in the serum than in the brain samples. LPC(18:1), LPC(22:6), LPC(16:0), LPC(14:0), LPC(17:0), LPC(18:0), and LPC(20:4) were 5-fold abundantly higher in the brain compared to the serum. LPC(20:4), LPC(14:0), and LPC(18:0) were also statistically significantly increased in the serum 3X (see Table 1).

### 2.4 Lipid Panels That Discriminate between Injury Severity

#### 2.4.1 Evaluation of Lipid Panel between Brain and Blood Repetitive (rmTBI) Groups

**Figure 5:**
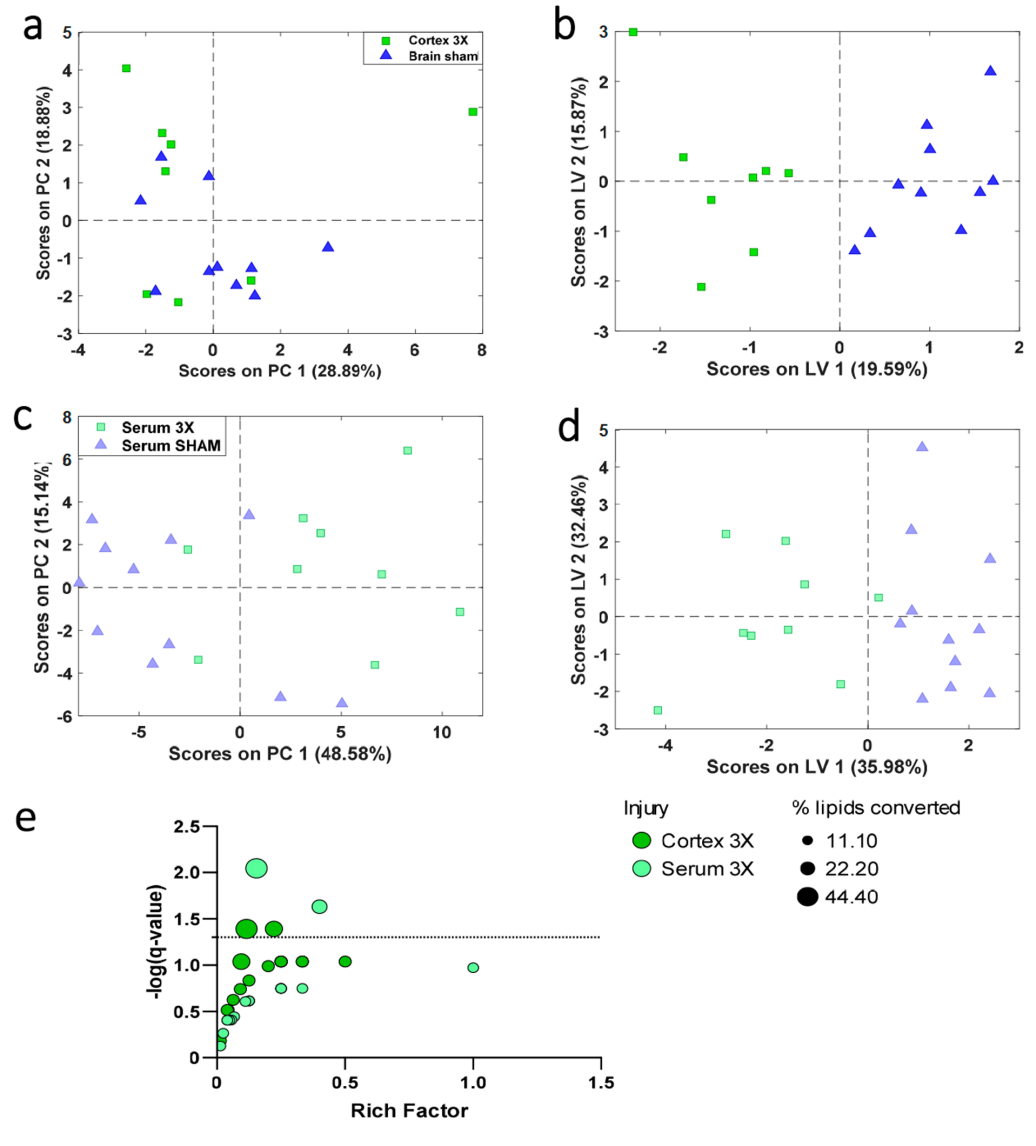
PCA and oPLS-DA models of brain and serum compartments comparing SHAM and rmTBI. (A) PCA score plot of 21 features in the brain that have high fold change values (± 1.5). (B) oPLSDA model of 11 features that distinguish between SHAM and 3X with a sensitivity of 87.5% and specificity of 100%. (C) PCA score plot of 69 features in the brain that have high fold change values (± 1.5). (D) oPLSDA model of 13 features that distinguish between SHAM and 3X with sensitivity 88.9% and specificity of 100%. Principal component analysis (PCA), orthogonal partial least-squares discriminant analysis (oPLS-DA). (E) LIPEA pathway analysis for lipids in final panels. The Rich factor, shown along the *x*-axis, represents the number annotated lipids belonging to a specific pathway in the data set out of total of known lipids in the pathway. The *y*-axis represents logarithmic value using the Benjamini–Hochberg correct factor. The size of bubbles represents the percentage of lipids converted from the final panel.

Among overlapping features with tentative annotations, there were 318 features in the cortex and 349 in the serum data sets (Figure S2). Redundant features remained for the analysis of the lipid panels that overlapped in both the cortex (Figure S3A) and the serum (Figure S3B) compartments. The data sets were further reduced by limiting fold change values of 1.5 between 3X and sham control, leaving 21 features in the cortex data set (Figure 5A) and 69 features in the serum data set (Figure 5B). The data indicate that there are more prominent changes in the serum compared to the cortex due to injury. Using this method to reduce features, there were only 3 lipids in the reduced cortex and serum data sets that made the fold change criteria and overlapped between raw data sets, which were Car(5:0), LPE(22:6), TG(46:2), and TG(46:1). A PCA score plot of the 21 features in the cortex illustrates minimal separation between the sham and 3X groups; therefore, other supervised classification methods were needed to further discriminate features. A genetic algorithm with Venetian blinds cross-validation was used to obtain a list of 11 features (Figure 5B and Table S2). oPLS-DA plot demonstrated discrimination of the 11 metabolites along PC1 with a high sensitivity and specificity of 100%. The final panel that discriminates between brain 3X and sham control samples contained lipids involved in different pathways (Table S3). It is noted that the TG(16:1_18:1_18:2) may not be a TG due its presence in negative mode, and we have tentatively annotated the data sets based on chemical formula and retention time. The same methods were used to reduce the serum features with a high fold change value of 1.5 from 69 to 13 lipids. Orthogonal partial least-squares discriminant analysis (oPLS-DA) plot showed the separation between serum 3X and sham control groups with a high sensitivity and specificity along PC1 (Figure 5D and Table S4) and the separation of male and female groups along PC2. The final panel that discriminates between serum 3X and sham control samples contains lipids involved in several pathways (Table S5). Lipid Enrichment Pathway Analysis (LIPEA) of the final brain and serum 3X panels shows overlapping of the alpha-linoleic acid metabolism, arachidonic acid metabolism, choline metabolism, glycerophospholipid metabolism, linoleic acid metabolism, retrograde endocannabinoid signaling, and sphingolipid metabolism and signaling pathways (Figure 5E).

#### 2.4.2 Evaluation of Lipid Panel between Brain and Blood Single mTBI (smTBI) Groups

**Figure 6:**
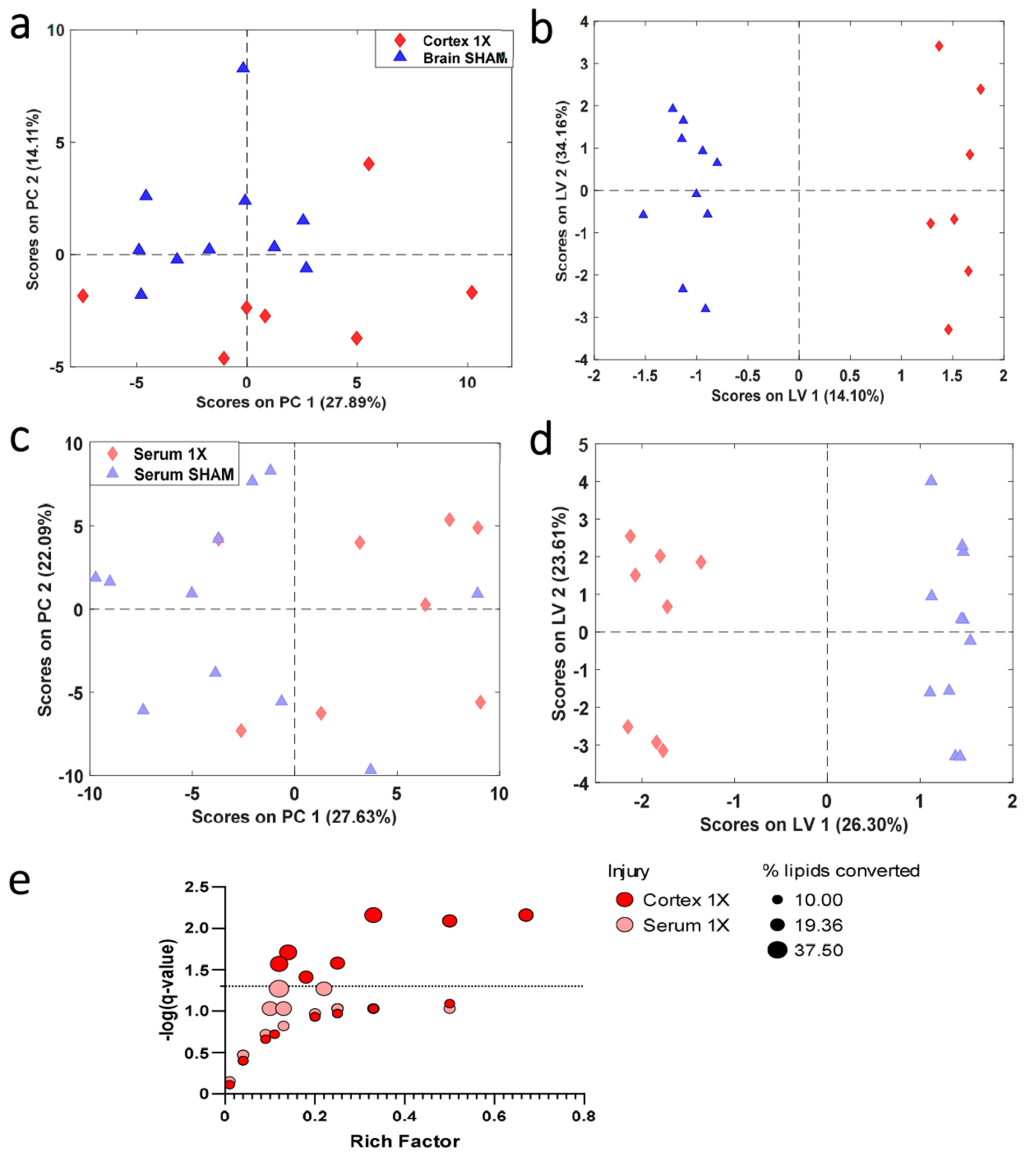
PCA and oPLS-DA score plots of cortex and serum compartments comparing SHAM and smTBI. (A) PCA score plot of 70 features in the brain that have high fold change values (± 1.20). (B) oPLS-DA model of 13 features that distinguish between SHAM and 1X with sensitivity and specificity of 100%. (C) PCA score plot of 136 features in the serum that have high fold change values (± 1.20). (D) oPLS-DA model of 12 features that distinguish between SHAM and 1X with sensitivity and specificity of 100%. Principal component analysis (PCA), orthogonal partial least-squares discriminant analysis (oPLS-DA). (E) LIPEA pathway analysis for lipids in final panels. The Rich factor, shown along the *x*-axis, represents the number annotated lipids belonging to a specific pathway in the data set out of total of known lipids in the pathway. The *y*-axis represents a logarithmic value using the Benjamini–Hochberg correct factor. The size of the bubbles represents the percentage of lipids converted from the final panel.

To evaluate lipids that discriminate between 1X and sham control samples in both the brain and serum compartment, similar methods were used (Figure 5). The compartments were further reduced by limiting to a fold change value of 1.20 between brain 1X and sham control and 1.20 between serum 1X and sham control. Examination of lipids that discriminate between brain sham and 1X injury groups revealed 70 annotated features following reduction based on a fold change of 1.20. There was a slight separation along PC2 for the cortex samples (Figure 6A). A genetic algorithm was used to further reduce features to a 13-feature panel that can discriminate between sham and 1X with a sensitivity and specificity of 100% (Figure 6B and Table S6). The final panel that discriminates between brain 1X and sham control samples contains lipids involved in various metabolic pathways (Table S7). The features in the serum 1X samples were reduced by fold change values of 1.20. The PCA score plot of the 136 annotated features illustrated minimal separation between sham and 1X injury serum groups using similar methods above (Figure 6C). A genetic algorithm was used to reduce features; the oPLS-DA score plot illustrates discrimination between sham and 1X injury groups with a sensitivity and specificity of 100% (Figure 6D and Table S8). The final panel that discriminates between the serum 1X and sham control samples contains lipids involved in various pathways (Table S9). Pathway analysis of the final brain and serum 1X panels indicated overlapping of the adipocytokine signaling, AGE-RAGE signaling, alpha-linoleic acid metabolism, autophagy, arachidonic acid metabolism, choline metabolism, ether lipid metabolism, glycerophospholipid metabolism, ferroptosis insulin resistance, Leishmaniasis, linoleic acid metabolism, necroptosis, neurotrophin signaling, retrograde endocannabinoid signaling, and sphingolipid signaling pathways.

## 3 Discussion

Brain and serum lipidome changes were investigated in female and male rats in the acute phase following a clinically relevant closed head mTBI model using ultra-high performance LC-MS and genetic algorithms for feature selection. We annotated lipids with an in-house data dictionary and focused on lipids that were detected in both the brain and serum compartments in a single and repetitive mTBI rat model. This work illustrates extensive lipidome changes in the cortex and serum at 24 h post-injury. Some alterations of the lipid subclasses were mirrored in the cortex and serum compartments, but other lipid subclasses demonstrated an inverse relationship. Also, there were differentially enriched lipids in the cortex and the serum, which may suggest efflux of some brain lipids to the peripheral blood after TBI. Cumulatively, our lipid panel identified lipids that discriminate between sham control and injury in both compartments separately and may be indicative of similar pathological processes in the brain and serum compartments as evidenced by overlapping pathways.

The 250 lipids annotated by LC-MS in the cortex and serum data sets suggest more changes in the blood compared to the brain due to mTBI. This may be due to different pathological time courses in the brain and the blood compartments or downstream peripheral effects of other organs that release lipids into the blood. Previous literature shows temporal changes in the brain during the acute phase after stroke. For example, a study investigated temporal profiles of sphingolipid after middle cerebral artery occlusion and found a decrease of SM at 3 h and an increase of Cer at 24 h in the brain.^48^ Another study used Desorption Electrospray Ionization – Mass Spectrometry Imaging (DESI-MSI) to investigate temporal profile of brain lipidome changes after cerebral ischemia and found significant changes in phospholipid, sphingolipid, and glycerophospholipid species in the brain as early as 3 h and continuing up to 48 h post-ischemia.^49^ There were both an increase and decrease of these lipids in the cortex and striatum. In the acute phase post-TBI—at 4 and 24 h—there was a decrease of PC(34:4).^50^ When looking at acute changes in the blood after TBI, our previous study showed changes in the serum compartment at 30 min and 4-h post-injury; however, changes were minimal at 30 min.^42^ There are limited studies investigating temporal profiles in the early acute phase. There is a need for further studies to elucidate lipid alterations and metabolomic injury responses over time.

A major finding from our study showed changes in the lipid subclass, where LPC, LPE, PE, PI, PS, and TGs were reflective in each compartment in both injury severities (1X and 3X). There were mixed results in the Car, Cer, PC, and SM subclasses, with either an increase in the serum and decrease in the brain in the single or repetitive mTBI models. Additionally, we also saw an inverse relationship in the DG and FA subclasses where the lipids increased in the serum and decreased in the brain for some metabolites. Studies have shown an increase in free fatty acid species after TBI. A previous study from our group has shown arachidonic acid and FFA(18:0) increased and FFA(18:2 + 1O) decreased in the sera at 3 days post-moderate TBI.^36^ Another study showed an increase of oxidized, anti-inflammatory, and pro-inflammatory free fatty acids in a pediatric, rat controlled cortical impact (CCI) model in the acute phase.^51^ Other studies have shown increases in serum and brain glycerolipids during the acute and subacute phases after TBI. A previous study found an increase of DG(22:6_18:1), DG(22:6_18:2), and DG(20:4_18:1) in the serum after moderate CCI^36^ and DG(40a:6) in the brain after CCI.^52^ Our results of the brain lipid changes may conflict with previous literature due to differences in injury model and other protocol details. Previous preclinical TBI studies have reported a decrease in phospholipids in the blood after TBI in the acute and chronic phases. One study reported a significant decrease of PC, PE, and PI in the plasma 3 months post-CCI.^53^ Another study investigated phospholipid changes and found significant decrease of LPC, LPE, PC, PE, and PI at 3, 12, and 24 months compared to 24-h post-CCI time point.^40^ There were no significant changes of the phospholipids at 24 h after injury. However, our results indicate most phospholipids increased in the serum single and repetitive mTBI models. Other studies have reported mixed results of sphingolipid changes in the serum after TBI. A previous study reported a significant decrease of SM (22:0) and SM (22:1) and significant increase of dihydrosphingomyelin DHSM(16:0) and DHSM(18:0) in the brain after blast injury.^54^ We saw an increase in SM (36:0) and Cer (42:1) in the serum, similar to another study which found an increase of these lipids in the plasma after stroke.^43^ A targeted approach was used to investigate sphingolipid changes due to injury and the authors found an increase of sphingomyelins (SM) in the plasma after 4, 24, and 48 h post-TBI.^43^ The mixed results in the literature of the lipid class changes after TBI may point to the involvement of various pathways that cause changes in lipid concentrations in the brain or blood. Also, there is limited identification of lipid species, and more efforts are needed for a better understanding of lipidome changes after TBI.. A targeted approach was used to investigate sphingolipid changes due to injury and the authors found an increase of sphingomyelins (SM) in the plasma after 4, 24, and 48 h post-TBI. We saw an increase in SM (36:0) and Cer (42:1) in the serum, similar to another study which found an increase of these lipids in the plasma after stroke.^43^ A targeted approach was used to investigate sphingolipid changes due to injury, and the authors found an increase of sphingomyelins (SM) in the plasma after 4, 24, and 48 h post-TBI.^43^ The mixed results in the literature of the lipid class changes after TBI may point to the involvement of various pathways that cause changes in lipid concentration in the brain or blood. Also, there is limited identification of lipid species, and more efforts are needed for a better understanding of lipidome changes after TBI.

Another major finding from our study resulted in 4 lipid panels that discriminate between injury and sham control in the brain and blood compartments with high sensitivity and specificity. There is evidence of some degree of overfitting of the models, which is due to the low sample size. Therefore, we make minimal claims of biological significance of candidate panels. To overcome this obstacle, there is a need for a larger cohort for future studies. The repetitive mTBI brain and serum panels contained lipids involved in overlapping pathways. The glycerophospholipid metabolism pathway was the only statistically significant pathway (*q* < 0.05) in both the brain and serum panels. However, there are no significant overlapping pathways between the brain and serum in the single mTBI group. This may be due to limited identification of lipid pathways in the LIPEA database due to only some lipids being mapped to pathways. Additionally, we acknowledge the need of further work to identify more specific signaling pathways that may lead to better understanding of lipidome changes in the brain and blood for TBI biomarker discovery. Phospholipids are important molecules in signaling and provide structural support to neural membranes. When evaluating lipids in the panels, there was a presence of Car (20:4) in both the repetitive brain and serum mTBI panel. Although Car(20:4) is not statistically significant in the panels, it may be any interesting molecule because of its presence in the final candidate panels. There is no current pathway analysis of Car(20:4) and its role in biological systems; however, the carnitines has been investigated in various biological processes. Acetyl-l-carnitine and l-carnitine, nonprotein amino acids, have been studied as therapeutic drugs. Specifically, a study previously demonstrated that acetylated derivative acetyl-l-carnitine (ALCAR) provided neuroprotective effects by providing acyl and fatty acid moieties and supplying energy for lipid synthesis^55^ in neurological diseases such as TBI.^1^ Studies have shown the administration ALCAR to rat pups after brain injury improved functional outcomes^56^ and behavioral outcomes in lesioned rat pups.^57^

Sphingolipids are highly enriched in the brain, especially the white matter, compared to plasma.^43^ Our results indicated that Cer(d18:1/18:0), HexCer(d18:1/24:1-OH), Cer(d18:0/22:0), SM(d36:0), SM(d36:2), Cer(d18:1/22:1), HexCer(d44:1-OH), sphingosine (C18), Cer(d18:1/23:1), and SM(d36:1) are highly abundant in the brain and relatively lower in the serum. A study found that SM(d36:1) and SM(d36:2), brain-specific lipids, were highly enriched in the brain compared to the plasma and other tissue compartments^44^ and increased in the serum due to cerebral ischemia.^43^ Our results show that these brain-specific lipids were over 3-fold higher in the brain compared to that in the serum. Additionally, there was a statistically significant increase in SM(d36:1) and SM(d36:2) in the serum in our repetitive mTBI group and no change in single mTBI, which may be due to more white matter damage with increased injury severity. We speculate that this lipid may efflux from the brain to the peripheral blood after TBI and has the potential to be a TBI biomarker.

This work investigated compartment lipidome dysregulation after mTBI, but there are limitations to the work. First, the lipid subclasses and class change we presented represented only a subset of the data due to limitations in identification of features in the complete data sets. Some of the trends of class lipid dysregulation after TBI are not expansive, and further studies are needed to fully elucidate class pattern changes after TBI. In addition, we used both male and female rats to understand global lipidome changes after TBI. There is pronounced separation along PC1 of male and female serum samples (see Figure S1), which continues to be prominent in the reduced features. However, we did not evaluate sex as a biological variable in our study to evaluate lipids as candidate biomarkers for TBI since we chose to focus on overlap between brain and blood compartments, and the degree of separation was different between compartments, with far less in brain compared to serum. More studies with larger sample sizes will be needed to investigate sex differences in the brain and serum compartments to better understand sexual dimorphism following TBI, which will likely affect lipid biomarker discovery. Furthermore, larger data sets are needed to overcome overfitting in the machine learning models and to correlate lipid changes to histopathological and behavioral outcomes to better understand the relationship between lipid dysregulation and TBI manifestation.

In summary, lipids hold promise to translate clinically as diagnostic markers for TBI and further understand neuropathology. Further studies are needed to validate the findings and the biological significance of lipid changes. This study lays the groundwork for lipid biomarker discovery in other fluid compartments, investigation of brain-region-specific lipid changes, and elucidation of the temporal pattern of lipid efflux from the brain to peripheral blood.

## 4 Materials and Methods

### 4.1 Injury Protocol

All procedures were performed in accordance with guidelines set forth in the Guide for the Care and Use of Laboratory Animals (U.S. Department of Health and Human Services, Washington, DC, USA, Pub no. 85–23, 1985) and were approved by the Georgia Institute of Technology Institutional Animal Care and Use Committee (protocol #A100188). Female (*n* = 16) and male (*n* = 10) Sprague–Dawley rats (8 weeks old; Charles River, Wilmington, MA, USA) weighing between 300 and 400 g were kept on 12 h reverse light-dark cycles, with food and water available ad libitum. Animals were randomly assigned by a random generator (https://www.random.org/lists/) to either sham procedure (*n* = 10), single impact (*n* = 8), and repetitive impacts (*n* = 8) groups.

A controlled cortical impact (CCI) device (Pittsburgh Precision Instruments, Pittsburgh, PA, USA) was modified by placing a 1 cm silicone stopper (Renovators Supply Manufacturing, Erving, MA, USA) on the standard CCI piston and used to induce single and repetitive closed-head impacts. Rats were anesthetized with isoflurane (induction: 5% isoflurane; maintenance: 3% isoflurane) and removed from anesthesia 30 s prior to closed head impacts. Rats were placed in prone position on 1 in. thick ethylene-vinyl acetate foam (McMaster-Carr, Elmhurst, IL, USA). The impacts were delivered at the midpoint between the bregma and lambda skull suture landmarks of the dorsal surface of the closed head. All mTBI groups received impacts from the piston at a velocity of 5 m/s. The single impact group received one injury with a 5 mm head displacement. The repeat impact group received a total of 3 injuries with 2 min intervals between impacts with head displacements 5 mm, 2 mm, and 2 mm. Sham animals received procedures identical to those of injured animals, excluding impacts. Righting latency was recorded as an acute neurological indicator of injury following the final impact. Results showed repetitive impact groups took significantly longer to right than sham control animals (Table S1). There was no difference between the single impact and sham control groups.

### 4.2 Sample Collection and Preparation

Approximately 200 μL of whole blood was collected from the tail artery with a 20-gauge vacuette needle (Greiner-One, Monroe, NC, USA) or alternatively the gingival vein at 0.5, and 4 h and the left ventricle with a syringe 24 h after TBI. The 24-h time point was used in the analysis for this paper.^42^ Blood samples coagulated at room temperature for 45 min and were centrifuged for 15 min at 4 °C and 2500 RCF. Brains were collected following transcardial perfusion with phosphate buffer (0.1 M, pH 7.4) 24 h post-TBI. Perfused whole brains were rapidly removed and flash frozen in an isopentane-methanol ice slurry. Pieces of parietal cortices (5 mm × 2 mm) were dissected by removing the subcortical structures including white matter and stored at −80 °C in microcentrifuge tubes. The frozen cortices were then frozen in liquid nitrogen and manually pulverized with a pestle and mortar submerged in liquid nitrogen and aliquoted in ∼10–30 mg tissue samples.

The serum and brain samples were thawed on ice prior to addition of solvent (IPA and Splash II Lipidomix in (1:3 v/v)) to separate lipids and small nonpolar metabolites. Serum and solvent (1:3 w/v) were vortexed for 10 s and centrifuged at 16,000*g* for 7 min. LC-MS grade water was used to prepare sample blanks, and pooled QC samples were prepared from 5 μL of aliquoted supernatant of all serum samples in the study. Samples were run in a randomized order over a consecutive 2.5 days of instrument time. QC samples were interleaved every 24 runs to account for batch effects over days of experiments. Brain and solvent (1:4 w/v), and liquid chromatography beads were placed in a homogenizer for 8 min and centrifuged at 16000*g* for 7 min. The supernatant was collected for LC-MS. Pooled quality control samples were formed from combining 6 μL aliquots of all brain sample extracts. Sample blanks were prepared with the same procedure except instead of a brain sample, 50 μL of LC-MS grade water was used.

### 4.3 Sample Analysis with Ultra-high Performance Liquid Chromatography–Mass Spectrometry (UPLC-MS)

Detailed UHPLC-MS methods were previously described.^42^ Samples were analyzed by using a Vanquish Horizon UHPLC instrument coupled to an ID-X Orbitrap Tribrid mass spectrometer operated in both positive and negative ion modes. Both ion modes used identical two-part mobile phases. Mobile phase A was a (40:60 v/v) water/ACN mixture, and mobile phase B was a (90:10 v/v) IPA/ACN mixture. Both mobile phases contained 0.1% formic acid and 10 mM ammonium formate. The stationary phase used for both ionization modes was a 2.1 mm × 50 mm Accucore C30 column with 2.1 μm particle size. Samples were randomized and analyzed over a scan range of 150–2000 *m*/*z*.

LC-MS/MS experiments were acquired using a data dependent acquisition (DDA) strategy to aid in compound identification. MS spectra were collected with a resolution of 30,000 and the dd-MS2 were collected at a resolution of 15,000 and an isolation window of 0.8 *m*/*z*. Precursors were activated by HCD and CID activation. Stepped normalized collision HCD energies of 15%, 30%, and 45% fragmented selected precursors in the collision cell and produced ions were detected in the orbitrap. Normalized CID energy of 45% fragmented and analyzed ions in the ion trap. Dynamic exclusion was set at 6 s and duty cycle was set to 1 s.

### 4.4 Data Processing

Raw LC-MS data was processed using Compound Discoverer version 3.0.0 software and the XCMS web-based application. Xcalibur software was used to identify the standards in blanks. Initial processing steps include retention time peak alignment between samples, peak detection, peak area integration, isotope peak grouping, adduct peak grouping, gap filling, and drift correction. Features eluting with the solvent front or having retention times below 0.75 min were removed from the data set to account for potential ion suppression effects. Sample ID No. 10 was removed after the quality control step.

### 4.5 Annotation, Feature Selection, and Lipid Enrichment Pathway Analysis

The data set was annotated using spectral matching to an in-house spectral library. The data set was filtered for features detected in the brain and plasma samples and autoscaled prior to building models. Welch’s *t* test was performed for each feature between sham and injury groups. Features were further reduced using a genetic algorithm in the PLS Toolbox package. Selected features were then used to build an oPLS-DA model. Abbreviated lipids were imported into the lipid pathway enrichment analysis (LIPEA) web-based tool with the *Rattus norvegicus* background.^58^

### 4.6 Statistical Analysis

An unpaired *t* test with Welch’s correction was used to compare sham and TBI groups and brain and blood groups. Data were analyzed using GraphPad Prism 8 and Matlab. Reported p values are multiplicity adjusted to account for multiple comparisons. For all cases, significance was defined as *p* < 0.05 (*) or *p* < 0.01 (**), *p* < 0.001 (***), or *p* < 0.0001 (****).

## Supporting Information Available

Figures and tables that further describe righting latency, PCA score plot of total features detected by LCMS in the cortex and serum, PCA score plot of brain and serum overlapping data sets, heatmaps of *p*-values of injured relative to sham control for the brain and serum, tables of pathway analysis of each lipid panel that discriminates between injured relative to sham control (PDF)

## ABBREVIATIONS

Car: carnitine
CE: cholesteryl ester
Cer: ceramide
DG: diacylglycerols
FFA: free fatty acid
HexCer: hexosylceramides
LPC: lysophosphatidylcholine
LPE: lysophosphatidylethanolamine
mTBI: mild traumatic brain injury
PC: phosphatidylcholine
PE: phosphatidylethanolamine
PI: phosphatidylinositol
PS: phosphatidylserine
rmTBI: repetitive mild traumatic brain injury
SM: sphingomyelin
smTBI: single mild traumatic brain injury
TBI: traumatic brain injury
TG: triacylglycerols
