# Supplementary material for: Comparing Brain and Blood Lipidome Changes following Single and Repetitive Mild Traumatic Brain Injury in Rats
Source: ACS Chem Neurosci. 2024 Jan 5;15(2):300–14. doi: 10.1021/acschemneuro.3c00603 (PMC10797623; doi:10.1021/acschemneuro.3c00603)
Supplement: Supplementary file 1 — cn3c00603_si_001.pdf [file cn3c00603_si_001.pdf]

Supporting Information

Comparing Brain and Blood Lipidome Changes following Single and Repetitive Mild Traumatic Brain Injury in Rats

Alexis N. Pulliam[1,3], Eric C. Gier[2,3], David A. Gaul[2,3], Samuel G. Moore[2,3], Facundo M. Fernández[2,3,*], Michelle C. LaPlaca[1,3,*]

1. Coulter Department of Biomedical Engineering, Georgia Institute of Technology/Emory University, Atlanta, GA 30332 (USA)
2. School of Chemistry and Biochemistry, Georgia Institute of Technology, Atlanta, GA 30332 (USA)
3. Petit Institute for Bioengineering and Bioscience, Georgia Institute of Technology, Atlanta, GA 30332, (USA)

Correspondence and requests for materials should be addressed to M.C.L. (email: michelle.laplaca@bme.gatech.edu).

Table S1. Righting latency

Injury severity	Sexes	Righting Latency (min)(mean ± SEM)
SHAM	N= 10;<br>Males= 5<br>Females= 5	2.39 ±0.456<br>2.56 ± 0.437<br>2.25 ± 0.790
1X	N= 8;<br>Males= 4<br>Females= 4	2.99 ± 0.342<br>3.76 ± 0.840<br>2.61 ± 0.224
3X	N= 8;<br>Males= 2<br>Females= 6	5.10 ± 1.078 *<br>6.09 ± 1.435<br>4.53 ± 1.519

Sample size of brain and serum samples collected. RmTBI rats took significantly longer to right than SHAM. * P<0.05 compared to SHAM of combined animals, Mixed-effect analysis was performed with Tukey's post-hoc test

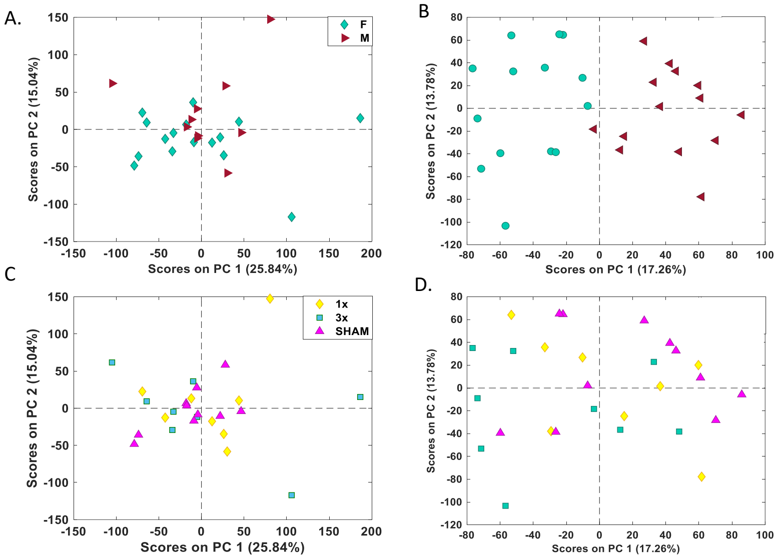

Figure S1. Features detected by LCMS in both brain and serum 24 hours post-mTBI. (**A**) PCA score plot of 14,909 features detected in the brain shows some overlap between sex. (**B**) PCA score plot of 14,119 features detected in serum shows clustering based on sex. Data show separation along PC1.(**C**) PCA scores plot of 14,909 features in the brain with various injury severities. Data shows minimal clustering between injury groups of the raw data set. (**D**) PCA score plots of 14,119 features detected in the serum show graded injury separation along the diagonal of PC1 and PC2.PC1 and PC2. F denotes female, M denotes male.

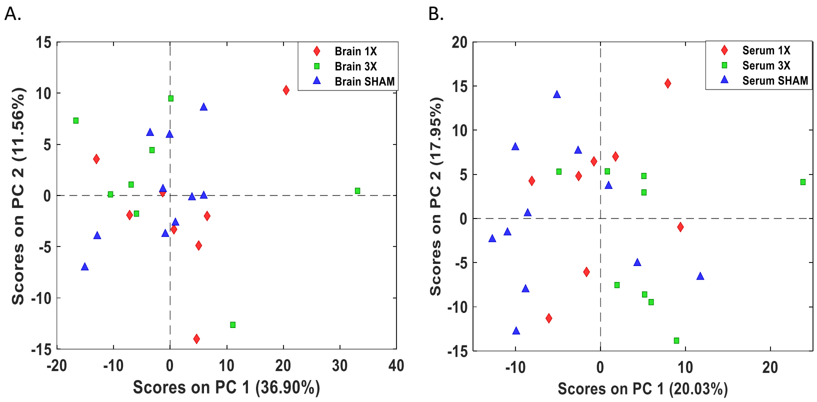

Figure S2. **Overlap of metabolites in the brain and serum compartments**. (**A**) PCA scores plot of 317 features in the brain with various injury severities. Data show minimal separation between injury groups. (**B**) PCA score plot of 348 features detected in the serum show minimal separation among injury groups.

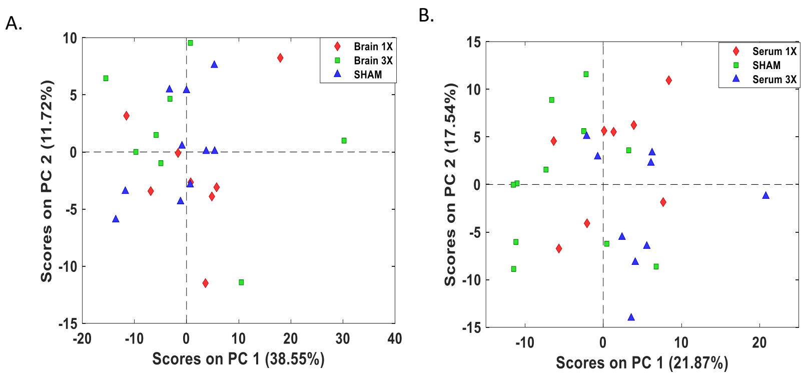

Figure S3. Aligned 250 features that overlap in the brain and serum compartments. (**A**) PCA scores plot of brain. Data show minimal separation between injury groups. (**B**) PCA score plot of serum show minimal separation among injury groups.

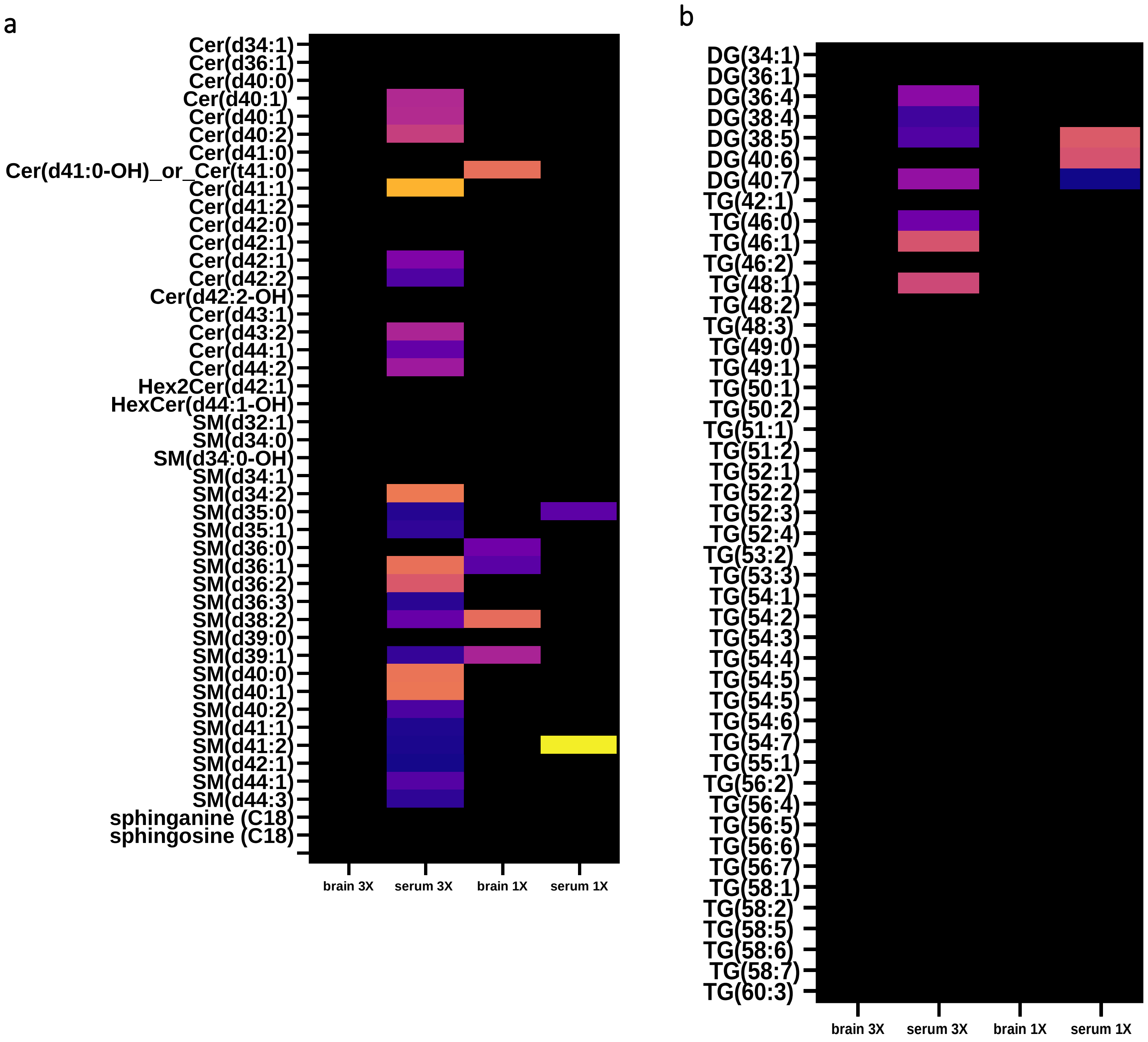
a
Cer(d34:1)
Cer(d36:1)
Cer(d40:0)
Cer(d40:1)
Cer(d40:1)
Cer(d40:2)
Cer(d41:0)
Cer(d41:0-OH)_or_Cer(t41:0)
Cer(d41:1)
Cer(d41:2)
Cer(d42:0)
Cer(d42:1)
Cer(d42:1)
Cer(d42:2)
Cer(d42:2-OH)
Cer(d43:1)
Cer(d43:2)
Cer(d44:1)
Cer(d44:2)
Hex2Cer(d42:1)
HexCer(d44:1-OH)
SM(d32:1)
SM(d34:0)
SM(d34:0-OH)
SM(d34:1)
SM(d34:2)
SM(d35:0)
SM(d35:1)
SM(d36:0)
SM(d36:1)
SM(d36:2)
SM(d36:3)
SM(d38:2)
SM(d39:0)
SM(d39:1)
SM(d40:0)
SM(d40:1)
SM(d40:2)
SM(d41:1)
SM(d41:2)
SM(d42:1)
SM(d44:1)
SM(d44:3)
sphinganine (C18)
sphingosine (C18)
brain 3X
serum 3X
brain 1X
serum 1X
b
DG(34:1)
DG(36:1)
DG(36:4)
DG(38:4)
DG(38:5)
DG(40:6)
DG(40:7)
TG(42:1)
TG(46:0)
TG(46:1)
TG(46:2)
TG(48:1)
TG(48:2)
TG(48:3)
TG(49:0)
TG(49:1)
TG(50:1)
TG(50:2)
TG(51:1)
TG(51:2)
TG(52:1)
TG(52:2)
TG(52:3)
TG(52:4)
TG(53:2)
TG(53:3)
TG(54:1)
TG(54:2)
TG(54:3)
TG(54:4)
TG(54:5)
TG(54:5)
TG(54:6)
TG(54:7)
TG(55:1)
TG(56:2)
TG(56:4)
TG(56:5)
TG(56:6)
TG(56:7)
TG(58:1)
TG(58:2)
TG(58:5)
TG(58:6)
TG(58:7)
TG(60:3)
brain 3X
serum 3X
brain 1X
serum 1X

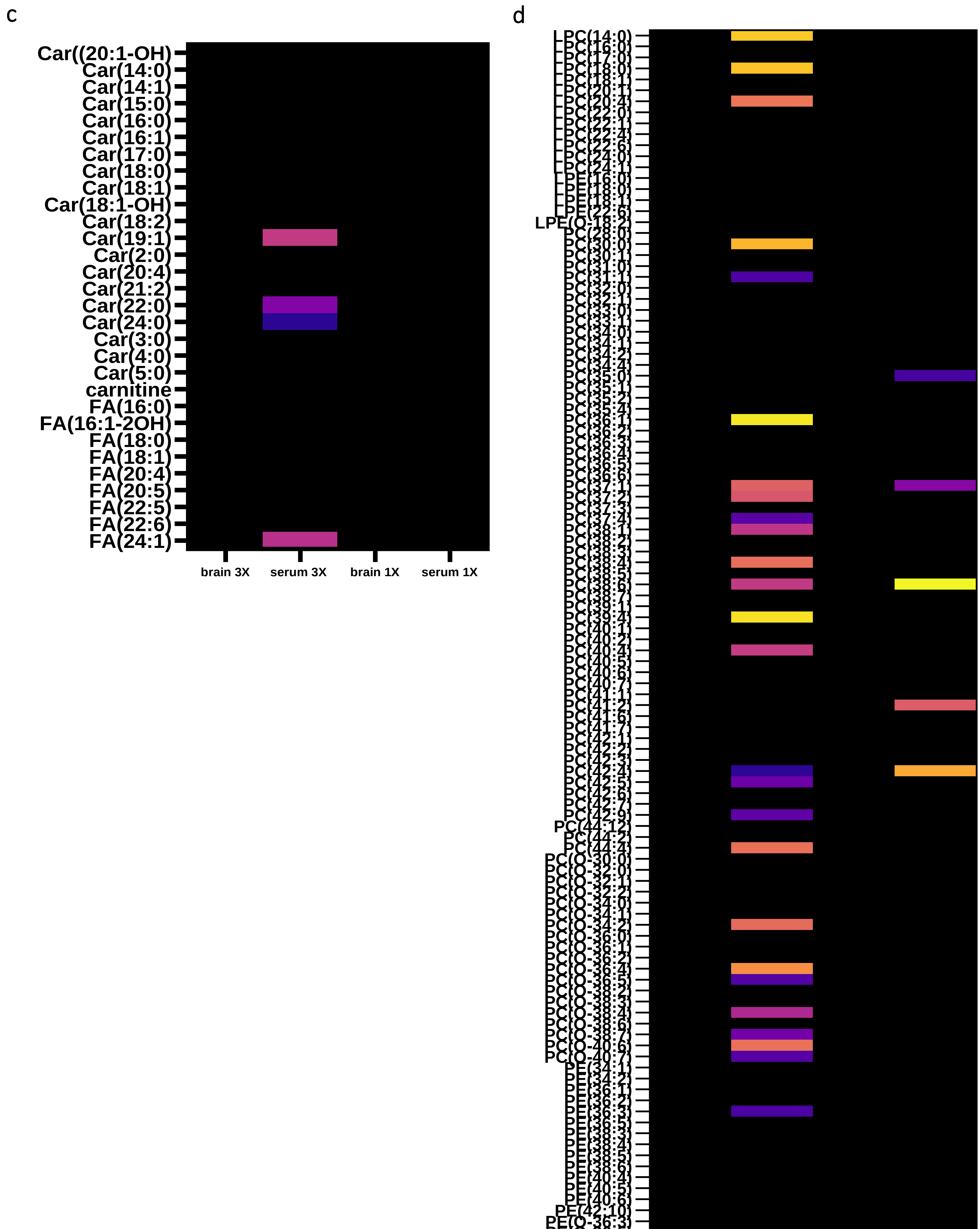

c
Car((20:1-OH)
Car(14:0)
Car(14:1)
Car(15:0)
Car(16:0)
Car(16:1)
Car(17:0)
Car(18:0)
Car(18:1)
Car(18:1-OH)
Car(18:2)
Car(19:1)
Car(2:0)
Car(20:4)
Car(21:2)
Car(22:0)
Car(24:0)
Car(3:0)
Car(4:0)
Car(5:0)
carnitine
FA(16:0)
FA(16:1-2OH)
FA(18:0)
FA(18:1)
FA(20:4)
FA(20:5)
FA(22:5)
FA(22:6)
FA(24:1)
brain 3X
serum 3X
brain 1X
serum 1X
d
LPC(14:0)
LPC(16:0)
LPC(17:0)
LPC(18:0)
LPC(18:1)
LPC(20:1)
LPC(20:4)
LPC(22:0)
LPC(22:1)
LPC(22:4)
LPC(22:6)
LPC(24:0)
LPC(24:1)
LPE(16:0)
LPE(18:0)
LPE(18:1)
LPE(22:6)
LPE(O-18:2)
PC(28:0)
PC(30:0)
PC(30:1)
PC(31:0)
PC(31:1)
PC(32:0)
PC(32:1)
PC(33:0)
PC(33:1)
PC(34:0)
PC(34:1)
PC(34:2)
PC(34:4)
PC(35:0)
PC(35:1)
PC(35:2)
PC(35:4)
PC(36:1)
PC(36:2)
PC(36:3)
PC(36:4)
PC(36:5)
PC(36:6)
PC(37:1)
PC(37:2)
PC(37:3)
PC(37:4)
PC(38:1)
PC(38:2)
PC(38:3)
PC(38:4)
PC(38:5)
PC(38:6)
PC(38:7)
PC(39:1)
PC(39:4)
PC(40:1)
PC(40:2)
PC(40:4)
PC(40:5)
PC(40:6)
PC(40:7)
PC(41:1)
PC(41:2)
PC(41:6)
PC(41:7)
PC(42:1)
PC(42:2)
PC(42:3)
PC(42:4)
PC(42:5)
PC(42:6)
PC(42:7)
PC(42:9)
PC(44:12)
PC(44:2)
PC(44:4)
PC(O-30:0)
PC(O-32:0)
PC(O-32:1)
PC(O-32:2)
PC(O-34:0)
PC(O-34:1)
PC(O-34:2)
PC(O-36:0)
PC(O-36:1)
PC(O-36:2)
PC(O-36:4)
PC(O-36:5)
PC(O-38:2)
PC(O-38:3)
PC(O-38:4)
PC(O-38:6)
PC(O-38:7)
PC(O-40:6)
PC(O-40:7)
PE(34:1)
PE(34:2)
PE(36:1)
PE(36:2)
PE(36:3)
PE(36:5)
PE(38:3)
PE(38:4)
PE(38:5)
PE(38:6)
PE(40:4)
PE(40:5)
PE(40:6)
PE(42:10)
PE(O-36:3)

Figure S4. Heatmaps of p-values between injured and sham control of lipid classes. The heatmaps depict p-values of only lipids that were statistically significant ($p<0.05$) (**A**) Sphingolipid (**B**) Glycerolipids (**C**) Free Fatty Acids (**D**) Phospholipids

Table S2. Lipid panel that discriminates between repetitive mTBI (rmTBI) and sham control groups in the brain

Feature Number	Annotation	Formula	m/z, mass error (ppm)	Retention time	3X vs SHAM (p-value)	Fold change	Mode
7769	Car(20:4)	C27 H45 N O4	447.3352<br>1.900	1.711	0.869	-0.590	Pos
11197	Car(5:0)	C12 H23 N O4	245.16272<br>2.284	0.805	0.197	-0.686	Pos
7999	PC(44:2)	C52 H100 N O8 P	897.72002<br>2.131	7.156	0.264	-1.307	Pos
8100	PE(O-18:1/22:5)	C45 H80 N O7 P	777.56816<br>1.888	5.505	0.055	-0.622	Pos
14411	TG(46:2)	C49 H93 N O6	791.70145<br>0.773	7.732	0.193	0.482	Pos
14421*	TG(16:1_18:1_18:2)	C55 H101 N O6	871.76435<br>1.045	8.082	0.957	-1.155	Neg
12628	Cer(34:2)	C44 H85 N O3	675.65361<br>1.795	7.379	0.030	1.067	Pos
7898	LPE(18:1)	C23 H46 N O7 P	479.3016<br>2.001	2.058	0.057	0.824	Pos
4853	LPE(22:6)	C27 H44 N O7 P	525.28594<br>1.807	1.683	0.233	0.801	Pos
14406	TG(42:1)	C45 H87 N O6	737.65439<br>2.168	7.398	0.145	1.050	Pos
14422	TG(53:3)	C56 H105 N O6	887.79411<br>0.527	8.453	0.277	4.392	Pos

Annotation of 11-lipid panel that differentiates between 3X injury and sham control in the brain. Retention time, observed exact mass with error, observed electrospray adduct, predicted elemental formula, *p*-values, fold change (FC), injury group vs. sham control, are reported. Positive FC values correspond to increased abundance due to injury, and negative FC values correspond to decreased abundance due to injury. * denotes tentative identification of molecule that may not belong to the TG subclass.

Table S3. **LIPEA pathway analysis of lipid panel that discriminates cortex 3X and sham control.**

Pathway name	Pathway lipids (number)	Converted lipids (number)	Converted lipids (percentage)	KEGG Converted lipids (list)	p-value	Benjamini correction	Bonferroni correction
Glycerophospholipid metabolism	26	3	42.85714286	C00157, C04438, C05973	0.003160158	0.040665859	0.050562523
Sphingolipid signaling pathway	9	2	28.57142857	C00195, C12126	0.005083232	0.040665859	0.081331717
Adipocytokine signaling pathway	3	1	14.28571429	C00195	0.038884537	0.091652639	0.622152595
Sphingolipid metabolism	21	2	28.57142857	C00195, C12126	0.027482194	0.091652639	0.439715103
Neurotrophin signaling pathway	3	1	14.28571429	C00195	0.038884537	0.091652639	0.622152595
Necroptosis	4	1	14.28571429	C00195	0.051554609	0.091652639	0.824873747
AGE-RAGE signaling pathway	2	1	14.28571429	C00195	0.026069664	0.091652639	0.417114629
Leishmaniasis	4	1	14.28571429	C00195	0.051554609	0.091652639	0.824873747
Insulin resistance	4	1	14.28571429	C00195	0.051554609	0.091652639	0.824873747
Choline metabolism in cancer	5	1	14.28571429	C00157	0.064081246	0.102529994	1
Retrograde endocannabinoid signaling	8	1	14.28571429	C00157	0.100814055	0.146638626	1
Ferroptosis	11	1	14.28571429	C21484	0.136304144	0.181738859	1
Ether lipid metabolism	16	1	14.28571429	C04475	0.192788027	0.237277572	1
alpha-Linolenic acid metabolism	23	1	14.28571429	C00157	0.266530023	0.304605741	1
Linoleic acid metabolism	25	1	14.28571429	C00157	0.286506885	0.305607344	1
Arachidonic acid metabolism	75	1	14.28571429	C00157	0.655583004	0.655583004	1

Pathway analysis of lipid panel that discriminates between cortex 3X and sham control. Pathway names, Pathway lipids; Number of lipids in pathway, Converted lipids; number of lipids converted from dataset to pathway, Converted lipids percentage; percentage of lipids converted from panel, KEGG Converted lipids, p-values, Benjamini correction, Bonferroni correction

Table S4. Lipid panel that discriminates between rmTBI and sham control groups in the blood

Feature Number	Annotation	Formula	m/z, mass error (ppm)	Retention time	1X vs SHAM (p-value)	Fold change (FC)	Mode
4021	Car(24:0)	C31 H61 N O4	511.46015, -0.8955	3.288	0.002	0.691	Pos
4507	CE(22:6)	C49 H79 N O2	713.61152, -0.1527	8.654	0.157	0.813	Pos
4299	DG(36:4)	C39 H71 N O5	633.53344, -0.5256	5.655	0.017	1.181	Pos
4248	LPC(24:0)	C32 H66 N O7 P	607.45792, -0.5251	3.427	0.019	0.644	Pos
11717	PC(42:5)	C50 H90 N O8 P	863.64054, -0.4794	5.57	0.056	0.753	Pos
6227	PC(44:12)	C52 H80 N O8 P	877.56244, -0.3008	3.362	0.222	1.020	Pos
10830	SM(d36:0)	C41 H85 N2 O6 P	732.61536, 0.3904	5.018	0.091	1.176	Pos
4652	PC(33:0)	C41 H82 N O8 P	747.57826, -0.1257	4.954	0.507	-0.675	Pos
11312	PS(38:4)	C44 H78 N O10 P	811.53633, -0.6814	4.41	0.128	-0.901	Pos
5057	TG(46:0)	C49 H97 N O6	795.73226, 0.1533	8.439	0.080	-0.875	Pos
5024	TG(46:1)	C49 H95 N O6	793.71654, 0.0655	8.083	0.206	-1.097	Pos
5384	TG(48:1)	C51 H99 N O6	821.74789, 0.1241	8.433	0.151	-0.783	Pos
5580	TG(49:1)	C52 H101 N O6	835.76335, -0.1065	8.628	0.321	-0.610	Pos

Annotation of 13-lipid panel that differentiates between 3X injury and sham control in the serum. Retention time, observed exact mass with instrumental error, observed electrospray adduct, predicted elemental formula, *p*-values, fold change (FC), injury group vs. sham control, are reported. Positive FC values correspond to increased abundance due to injury, and negative FC values correspond to decreased abundance due to injury.

Table S5. **LIPEA pathway analysis of lipid panel that discriminates serum 3X and sham control.**

Pathway name	Pathway lipids (number)	Converted lipids (number)	Converted lipids (percentage)	KEGG Converted lipids (list)	p-value	Benjamini correction	Bonferroni correction
Glycerophospholipid metabolism	26	4	44.44444444	C04230, C02737, C00157, C04233	0.000474895	0.009023008	0.009023008
Choline metabolism	5	2	22.22222222	C04230, C00157	0.002463663	0.023404802	0.046809604
Systemic lupus erythematosus	1	1	11.11111111	C02737	0.016853933	0.106741573	0.320224719
Glycine, serine and threonine metabolism	3	1	11.11111111	C02737	0.049806225	0.17890184	0.946318275
Necroptosis	4	1	11.11111111	C00550	0.065911204	0.17890184	1
Leishmaniasis	4	1	11.11111111	C02737	0.065911204	0.17890184	1
Amoebiasis	4	1	11.11111111	C02737	0.065911204	0.17890184	1
Retrograde endocannabinoid signaling	8	1	11.11111111	C00157	0.127932166	0.243071115	1
Cholesterol metabolism	8	1	11.11111111	C02530	0.127932166	0.243071115	1
Fat digestion and absorption	8	1	11.11111111	C02530	0.127932166	0.243071115	1
Sphingolipid signaling pathway	9	1	11.11111111	C00550	0.142853479	0.246746918	1
Vitamin digestion and absorption	15	1	11.11111111	C02530	0.227711632	0.360543417	1
Ovarian steroidogenesis	18	1	11.11111111	C02530	0.267270792	0.390626542	1
alpha-Linolenic acid metabolism	23	1	11.11111111	C00157	0.329213206	0.394145369	1
Linoleic acid metabolism	25	1	11.11111111	C00157	0.352656382	0.394145369	1
Sphingolipid metabolism	21	1	11.11111111	C00550	0.305018397	0.394145369	1
Bile secretion	25	1	11.11111111	C02530	0.352656382	0.394145369	1
Steroid biosynthesis	41	1	11.11111111	C02530	0.515505556	0.544144753	1
Arachidonic acid metabolism	75	1	11.11111111	C00157	0.746718662	0.746718662	1

Pathway analysis of lipid panel that discriminates between serum 3X and sham control. Pathway names, Pathway lipids; Number of lipids in pathway, Converted lipids; number of lipids converted from dataset to pathway, Converted lipids percentage; percentage of lipids converted from panel, KEGG Converted lipids, p-values, Benjamini correction, Bonferroni correction

Table S6. Lipid panel that discriminates between single (smTBI) and sham control groups in the brain

Feature Number	Annotation	Formula	m/z, mass error (ppm)	Retention time	1X vs SHAM (p-value)	Fold change (FC)	Mode
7769	Car(20:4)	C27 H45 N O4	447.3352, 1.900	1.711	0.141	-0.794	Pos
14400	carnitine	C7 H15 N O3	161.1051, 2.824	0.889	0.990	-3.199	Pos
12628	Cer(d44:2)	C44 H85 N O3	675.6536, 1.795	7.379	0.198	0.580	Pos
7822	DG(18:0_22:6_0:0)	C43 H75 N O5	685.56536, 2.019	6.211	0.104	-0.362	Pos
7969	PC(40:7)	C48 H82 N O8 P	831.57904, 2.144	3.779	0.212	0.329	Pos
7991	PC(42:9)	C50 H82 N O8 P	855.57837, 1.301	3.512	0.394	0.498	Pos
8026	PC(O-38:3)	C46 H88 N O7 P	797.6309, 2.016	5.913	0.385	0.566	Pos
8082	PE(O-36:5)	C41 H74 N O7 P	723.5222, 3.397	4.615	0.451	0.494	Pos
8139	SM(d36:0)	C41 H85 N2 O6 P	732.61517, 1.630	4.91	0.012	0.418	Pos
8163	sphingosine	C18 H37 N O2	299.28268, 2.670	1.711	0.065	0.537	Pos
14410	TG(46:2) M + NH3	C49 H93 N O6	791.70122, 1.868	7.647	0.044	-1.747	Pos

Annotation of 11-lipid panel that differentiates between 1X injury and sham control in the brain. Retention time, observed exact mass with instrumental error, observed electrospray adduct, predicted elemental formula, *p*-values and fold change (FC), injury group vs. sham control, are reported. Positive FC values correspond to increased abundance due to injury, and negative FC values correspond to decreased abundance due to injury.

Table S7. LIPEA pathway analysis of lipid panel that discriminates brain 1X and sham control.

Pathway name	Pathway lipids (number)	Converted lipids (number)	Converted lipids (percentage)	KEGG Converted lipids (list)	p-value	Benjamini correction	Bonferroni correction
Glycosylphosphatidylinositol (GPI)-anchor biosynthesis	3	2	20	C00350, C01194	0.000939	0.006887	0.020661
Sphingolipid signaling pathway	9	3	30	C00195, C00550, C12126	0.000376	0.006887	0.008278
Autophagy - other	3	2	20	C00350, C01194	0.000939	0.006887	0.020661
Autophagy - animal	4	2	20	C00350, C01194	0.001859	0.008181	0.040907
Necroptosis	4	2	20	C00550, C00195	0.001859	0.008181	0.040907
Sphingolipid metabolism	21	3	30	C12126, C00195, C00550	0.005282	0.019367	0.116199
Retrograde endocannabinoid signaling	8	2	20	C00157, C00350	0.008334	0.026193	0.183354
Glycerophospholipid metabolism	26	3	30	C00350, C01194, C00157	0.009817	0.026996	0.21597
Ferroptosis	11	2	20	C21481, C21480	0.015883	0.038825	0.349428
AGE-RAGE signaling pathway in diabetic complications	2	1	10	C00195	0.037137	0.081701	0.817013
Neurotrophin signaling pathway	3	1	10	C00195	0.055236	0.093476	1
Adipocytokine signaling pathway	3	1	10	C00195	0.055236	0.093476	1
Kaposi's sarcoma-associated herpesvirus infection	3	1	10	C00350	0.055236	0.093476	1
Insulin resistance	4	1	10	C00195	0.073028	0.107108	1
Leishmaniasis	4	1	10	C00195	0.073028	0.107108	1
Tuberculosis	5	1	10	C01194	0.090518	0.117141	1
Choline metabolism in cancer	5	1	10	C00157	0.090518	0.117141	1
Inositol phosphate metabolism	9	1	10	C01194	0.157547	0.192558	1
Phosphatidylinositol signaling system	11	1	10	C01194	0.189365	0.219265	1
alpha-Linolenic acid metabolism	23	1	10	C00157	0.3586	0.39446	1
Linoleic acid metabolism	25	1	10	C00157	0.383482	0.401743	1
Arachidonic acid metabolism	75	1	10	C00157	0.782902	0.782902	1

Pathway analysis of lipid panel that discriminates between cortex 3X and sham control. Pathway names, Pathway lipids; Number of lipids in pathway, Converted lipids; number of lipids converted from dataset to pathway, Converted lipids percentage; percentage of lipids converted from panel, KEGG Converted lipids, p-values, Benjamini correction, Bonferroni correction

Table S8. Lipid panel that discriminates between single (smTBI) and sham control groups in the blood

Feature Number	Annotation	Formula	m/z, mass error (ppm)	Retention time	3X vs SHAM (p-value)	Fold change	Mode
3884	Car(20:4)	C27 H45 N O4	447.33498, 1.4978	1.718	0.698	-0.303	Pos
9290	Carnitine	C7 H15 N O3	161.10521, 3.507	0.792	0.028	0.347	Pos
4089	Cer(d34:1)	C34 H67 N O3	537.51229, 1.384	4.897	0.519	0.382	Pos
4299	DG(36:4)	C39 H71 N O5	633.53344, 1.206	5.655	0.084	0.291	Pos
4432	DG(18:1_22:6_0:0)	C43 H73 N O5	683.54915, 1.205	5.465	0.000	0.595	Pos
1033	LPE(22:6)	C27 H44 N O7 P	525.28685, 1.451	1.829	0.218	0.721	Neg
4574	PC(32:1)	C40 H78 N O8 P	731.54899, 4.147	3.535	0.489	0.273	Pos
4688	PC(34:4)	C42 H76 N O8 P	753.53117, 1.147	3.533	0.477	0.305	Pos
4844	PC(35:0)	C43 H86 N O8 P	775.60965, 1.409	5.992	0.033	0.403	Pos
4858	PC(36:6)	C44 H76 N O8 P	777.53093, 0.803	3.412	0.258	0.368	Pos
5115	PC(37:1)	C45 H88 N O8 P	801.62516, 1.189	6.077	0.016	0.387	Pos
5598	PC(40:4)	C48 H88 N O8 P	837.62469, 0.577	5.459	0.270	0.567	Pos
5573	PC(40:5)	C48 H86 N O8 P	835.60928, 0.865	4.592	0.915	-0.437	Pos
5515	PC(40:7)	C48 H82 N O8 P	831.57777, 0.617	3.74	0.913	-0.276	Pos
5798	PC(41:1)	C49 H96 N O8 P	857.68767, 1.006	6.952	0.138	0.492	Pos
5677	PC(41:7)	C49 H84 N O8 P	845.59372, 0.961	4.337	0.082	0.453	Pos
5838	PC(42:6)	C50 H88 N O8 P	861.62489	5.547	0.109	0.468	Pos
4811	PC(O-36:2)	C44 H86 N O7 P	771.61472, 1.397	6.067	0.041	0.365	Pos
4558	PE(O-36:3)	C41 H78 N O7 P	727.55201, 1.330	5.543	0.229	0.274	Pos

Annotation of 19- lipid panel that differentiates between 1X injury and sham control in the serum. Retention time, observed exact mass with instrumental error, observed electrospray adduct, predicted elemental formula, *p*-values, fold change (FC), injury group vs. sham control, are reported. Positive FC values correspond to increased abundance due to injury, and negative FC values correspond to decreased abundance due to injury.

Table S9. LIPEA pathway analysis of lipid panel that discriminates serum 1X and sham control.

Pathway name	Pathway lipids (number)	Converted lipids (number)	Converted lipids (percentage)	KEGG Converted lipids (list)	p-value	Benjamini correction	Bonferroni correction
Glycerophospholipid metabolism	26	3	37.5	C00157, C04438, C05973	0.004892514	0.053744833	0.078280228
Sphingolipid signaling pathway	9	2	25	C00195, C12126	0.006718104	0.053744833	0.107489665
alpha-Linolenic acid metabolism	23	1	12.5	C00157	0.298541047	0.341189768	1
Neurotrophin signaling pathway	3	1	12.5	C00195	0.044355783	0.094005497	0.709692523
Arachidonic acid metabolism	75	1	12.5	C00157	0.704598706	0.704598706	1
Ferroptosis	11	1	12.5	C21484	0.154331951	0.189947017	1
Sphingolipid metabolism	21	2	25	C00195, C12126	0.035777961	0.094005497	0.57244737
Linoleic acid metabolism	25	1	12.5	C00157	0.320353807	0.341710727	1
Retrograde endocannabinoid signaling	8	1	12.5	C00157	0.114463937	0.152618582	1
Necroptosis	4	1	12.5	C00195	0.058753436	0.094005497	0.940054971
Adipocytokine signaling pathway	3	1	12.5	C00195	0.044355783	0.094005497	0.709692523
Ether lipid metabolism	16	2	25	C05212, C04475	0.021236336	0.094005497	0.339781381
AGE-RAGE signaling pathway in diabetic complications	2	1	12.5	C00195	0.029765795	0.094005497	0.476252714
Leishmaniasis	4	1	12.5	C00195	0.058753436	0.094005497	0.940054971
Choline metabolism in cancer	5	1	12.5	C00157	0.072960931	0.106124991	1
Insulin resistance	4	1	12.5	C00195	0.058753436	0.094005497	0.940054971

Pathway analysis of lipid panel that discriminates between serum 1X and sham control. Pathway names, Pathway lipids; Number of lipids in pathway, Converted lipids; number of lipids converted from dataset to pathway, Converted lipids percentage; percentage of lipids converted from panel, KEGG Converted lipids, p-values, Benjamini correction, Bonferroni correction
